# Highlighting Human Enzymes Active in Different Metabolic Pathways and Diseases: The Case Study of EC 1.2.3.1 and EC 2.3.1.9

**DOI:** 10.3390/biomedicines8080250

**Published:** 2020-07-29

**Authors:** Giulia Babbi, Davide Baldazzi, Castrense Savojardo, Pier Luigi Martelli, Rita Casadio

**Affiliations:** Biocomputing Group, University of Bologna, 40126 Bologna, Italy; giulia.babbi3@unibo.it (G.B.); davide.baldazzi8@unibo.it (D.B.); castrense.savojardo2@unibo.it (C.S.); pierluigi.martelli@unibo.it (M.P.L.)

**Keywords:** enzymes, KEGG pathways KEGG metabolic pathways, protein-protein interaction, protein variation, protein stability

## Abstract

Enzymes are key proteins performing the basic functional activities in cells. In humans, enzymes can be also responsible for diseases, and the molecular mechanisms underlying the genotype to phenotype relationship are under investigation for diagnosis and medical care. Here, we focus on highlighting enzymes that are active in different metabolic pathways and become relevant hubs in protein interaction networks. We perform a statistics to derive our present knowledge on human metabolic pathways (the Kyoto Encyclopaedia of Genes and Genomes (KEGG)), and we found that activity aldehyde dehydrogenase (NAD(+)), described by Enzyme Commission number EC 1.2.1.3, and activity acetyl-CoA C-acetyltransferase (EC 2.3.1.9) are the ones most frequently involved. By associating functional activities (EC numbers) to enzyme proteins, we found the proteins most frequently involved in metabolic pathways. With our analysis, we found that these proteins are endowed with the highest numbers of interaction partners when compared to all the enzymes in the pathways and with the highest numbers of predicted interaction sites. As specific enzyme protein test cases, we focus on Alpha-Aminoadipic Semialdehyde Dehydrogenase (ALDH7A1, EC 2.3.1.9) and Acetyl-CoA acetyltransferase, cytosolic and mitochondrial (gene products of ACAT2 and ACAT1, respectively; EC 2.3.1.9). With computational approaches we show that it is possible, by starting from the enzyme structure, to highlight clues of their multiple roles in different pathways and of putative mechanisms promoting the association of genes to disease.

## 1. Introduction

It is common knowledge that enzymes are proteins characterized by specific molecular functions that, when performed in a concerted manner, give rise to the richness of biological processes at the basis of the cell complex physiology [1]. It is still a matter of debate whether different enzyme molecules tend to transiently aggregate in the cell environment, for generating the proper concerted action [2], and references therein. In the case of enzymes, any concerted biological process is modelled by a metabolic network/pathway that describes the biochemical sequential interactions and/or cycles at the basis of the cell metabolism [3]. Information on which models of metabolic pathways and reactions are known in a specific organism is also available through curated databases, such as the Kyoto Encyclopaedia of Genes and Genomes (KEGG) and REACTOME [4,5]. Each enzyme is a protein molecule endowed with a specific four-digit EC number [6], which fully describes the catalyzed biochemical reaction, and possibly with an atomic solved structure, routinely available in the Protein Data Bank (PDB), [7]. This allows for an understanding of the relationship between sequence, structure, and function at the basis of the catalytic mechanisms at the active site/s and the role of possible effectors at the binding site/s. UniProt/SwissProt [8] is the reference database for sequences, and PDB for three dimensional (3D) structures. Many enzymes are known to be involved in genetic diseases, as reported in OMIM (Online Mendelian Inheritance in Man) [9], as well as somatic diseases, including cancers (BioMuta [10], DisGenNet [11], Clinvar [12], MalaCards [13], etc.). This makes it possible to derive information on specific molecular mechanisms when non-synonymous mutations have been associated to specific pathologies. Thanks to massive proteomic experiments, we also know partners of interactions in the cell milieu stored in databases such as IntAct [14] and BioGRID [15]. Several databases are presently available for enzyme complete functional annotation, including BRENDA [16], Enzyme Portal (EBI) [17], and M-CSA (EBI) [18]. Furthermore, among other information, available data on the extent of expression of the enzymes in the different human tissues can be found in GeneCards [19].

The more data accumulated, the more we need linking different databases in order to derive general rules of molecular functioning, which reconcile molecular mechanisms to physiological models related to specific phenotypes. A recently released version of Manet (Molecular Ancestry Network, Manet 3.0, [20,21]) groups enzymatic activities into a hierarchical system of subnetworks and mesonetworks matching KEGG classification and including structural data.

Focusing on humans, here, we ask the question of how many human enzymes are common to different metabolic pathways. The aim is highlighting the complex networks of networks where some of the proteins are involved simultaneously in different biological processes and providing evidence of possible associations to protein-protein interaction data and molecular clues.

By referring to the human KEGG metabolic maps, we provide a list of these enzymes, and their relation to maladies, when known. We find an interesting correspondence among most frequent enzymes in KEGG metabolic maps, number of interactors in the cell environment and number of predicted interaction sites.

We then investigate, at a molecular level, one of these enzymes, ALDH7A1, a member of subfamily 7 in the aldehyde dehydrogenase gene family (EC 1.2.1.3). The enzymes are described to play a major role in the detoxification of aldehydes generated by alcohol metabolism and lipid peroxidation. The protein, characterized by at least three different isoforms, is present in the cytosol, the mitochondrion, and the nucleus, and it is associated with different biological functions. By means of computational tools, we investigate which structural properties of the enzyme can be indicative of its important role and highlight possible mechanisms of its failure, associated mainly with pyridoxine-dependent epilepsy (PDE). Similarly, we describe molecular experimental and predicted details of ACAT1 and ACAT2, performing in humans Acetyl-CoA C-acetyltransferases activity, respectively in the cytosol and in mitochondria (EC 2. 3.1.9). 

## 2. Experimental Section

### 2.1. Materials

For our analysis, we derived information from SwissProt/UniProt. Presently, SwissProt (release 04_2019) lists 20,365 human proteins, among which 3428 are enzymes specified with a complete enzyme commission number (EC with four digits, describing the biochemical reaction as to substrate and product) [6]. In the following, we will refer to enzyme proteins as EC proteins. We associate 7316 proteins with genetic diseases through our database eDGAR (adopting OMIM, HUMSAVAR, CLINVAR, and curated DisGeNet as primary sources of information) [22,23]. We find that 1699 proteins are EC proteins with associations to disease (Table 1).

For KEGG pathway annotation, we adopted the April 2020 KEGG release [4], with the distinction among KEGG pathways and KEGG metabolic pathways and with reference to human genes. Protein–protein interactions are retrieved from IntAct ([14], release June 2020) and BioGRID ([15], release 3.5.185).

### 2.2. Computational Methods

The likelihood of a protein lateral side chain to interact with other proteins is computed with ISPRED4 (Interaction Site PREDictions, version 4) [24,25], a machine-learning-based predictor performing at the state of the art. It predicts the interaction sites from protein structure with an accuracy as high as 85% and with a very low rate of false positive prediction (3%). When a structure is not available, an in-house version of ISPRED4 considering only sequence information is adopted. For computing the effect on protein stability of missense variations, we adopted INPS (Impact of Non synonymous variations on Protein Stability) [26,27]. Starting from information extracted from protein structure or sequence, INPS performs a non-linear regression based on machine learning approaches and reaches a Pearson’s correlation coefficient as high as 0.76 (0.71 when a structure is not available). The computed ∆∆G values have an associated standard error of about 1 kcal/mol.

## 3. Results

### 3.1. EC Proteins and KEGG Metabolic Pathways

In order to cope with the complexity of the network of human biochemical reactions, we focused on the analysis of all the possible relationships among biological functions as described by EC numbers and KEGG pathways. The Kyoto Encyclopaedia of Genes and Genomes (KEGG), [4], includes 320 biological pathways, 90 of which are specifically termed metabolic pathways. We annotated EC human proteins with KEGG terms for pathways (Table 2). Having as a reference the human protein section of SwissProt, we find that 6904 proteins are associated with 320 KEGG pathways. When focusing on proteins associated with metabolic KEGG pathways, 1642 EC proteins participate into 90 metabolic pathways. Restricting to proteins that are enzymes and disease-related, we obtained 770 EC proteins associated with 90 metabolic pathways. The 770 proteins are associated with 930 EC numbers.

Not all the EC proteins in SwissProt are associated with KEGG metabolic pathways (883 from Table 2). The whole network model is therefore complicated [20,21], and here, we focus only on KEGG networks that describe metabolic pathways.

**Figure 1:**
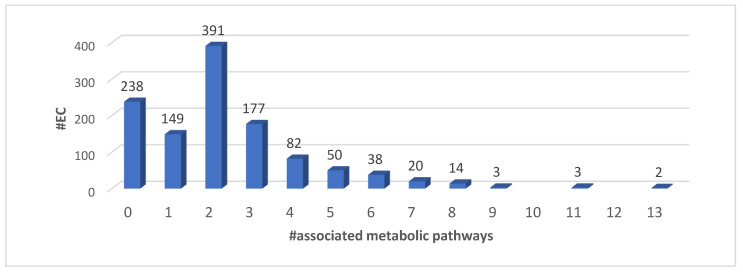
Distribution of functional activities (four-digit EC numbers) as a function of KEGG metabolic pathways.

In Figure 1, we show the distribution of EC numbers (which we consider here the complete description of the protein molecular activity) in the KEGG metabolic pathways. We find that five EC numbers are involved in at least 11 metabolic pathways—EC 1.2.1.3, Aldehyde dehydrogenase (NAD+); EC 1.14.14.1, Unspecific monooxygenase; EC 2.3.1.9, Acetyl-CoA C-acetyltransferase; EC 2.6.1.1, Aspartate transaminase; EC 4.2.1.17, Enoyl-CoA hydratase.

**Figure 2:**
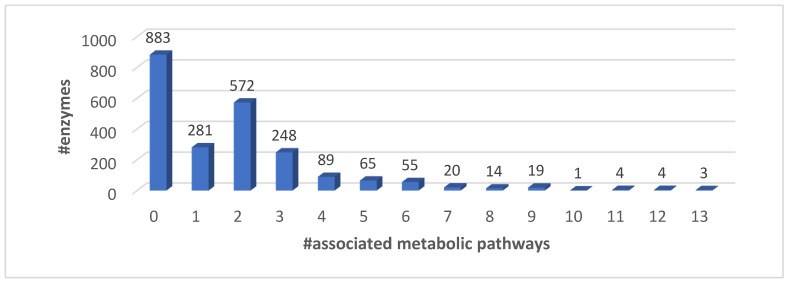
Distribution of EC proteins as a function of KEGG metabolic pathways.

The correspondence among EC numbers and proteins is plurivalent (an EC may be associated with different proteins and a protein with different ECs). The EC proteins to KEGG metabolic pathways association is shown in Figure 2.

The distribution of the EC proteins in the different KEGG metabolic pathways indicates that only 12 EC proteins are associated with 10 or more KEGG metabolic pathways (Table 3). The most frequent activities associated with the most frequent EC proteins are one oxidoreductase: EC 1.2.1.3 (aldehyde dehydrogenase (NAD+); two transferases: EC 2.3.1.9 (Acetyl-CoA C-acetyltransferase), EC 2.6.1.1 (Aspartate transaminase); and one lyase: EC 4.2.1.17 (enoyl-CoA hydratase). For details on the specific biochemical reactions including the description of substrates and products, refer to the Rhea database [28].

### 3.2. EC Proteins and Their Interactions

**Figure 3:**
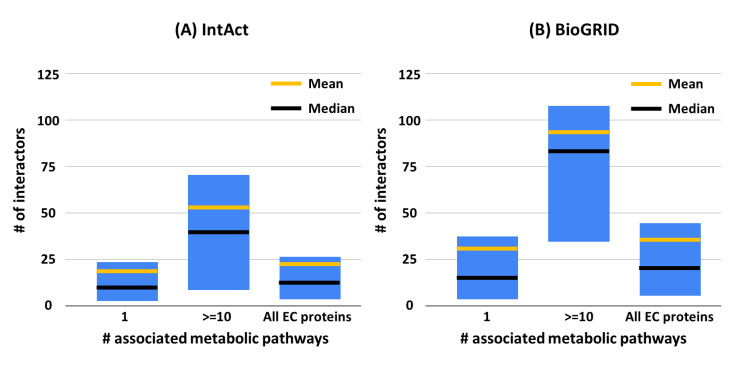
Statistical characterization of the number of interactors in EC proteins associated with human metabolic pathways. For each set, the boxes represent the first and third quartiles; yellow and black lines represent mean and median values, respectively. (**A**) and (**B**): from IntAct [14] and BioGRID [15], respectively. Significance of the reported differences on median values has been validated with the Mann–Whitney *U* test, obtaining *p*-value < 0.0001 when comparing the EC proteins with at least 10 interactors with the other two classes, for both IntAct and BioGRID databases. # Number of.

A network of networks can model all the interactions that each protein can have. To exploit this possibility in the light of the available results, we focused on the EC proteins that are associated with 10 or more KEGG metabolic pathways to highlight the number of their possible interactors (Figure 3). Experimental and physical interactions are retrieved from IntAct [14] and BioGRID [15]. When restricting to interactions among human proteins, IntAct reports 337,389 interactions among 36,815 proteins (including isoforms) and BioGRID reports 471,774 interactions involving 25,420 proteins. The average number of interactors per protein is therefore equal to 18 and 37 in IntAct and BioGRID, respectively. In Figure 3, the characteristic values (average, median, first and third quartiles) of the distribution of the number of interactors reported in IntAct and BioGRID are compared among the following classes—(i) proteins involved in only one metabolic KEGG, (ii) proteins involved in at least ten metabolic KEGG pathways, and (iii) all EC proteins. EC proteins involved in a high number of KEGG metabolic pathways have also a high number of interactors, when compared to those less frequently involved.

**Figure 4:**
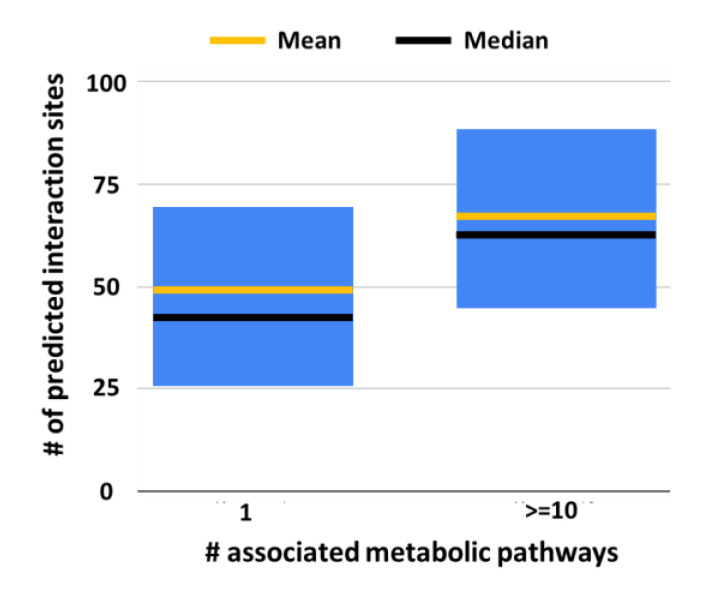
Statistical characterization of the number of interaction sites predicted with ISPRED4 in EC proteins associated with only one or at least 10 metabolic pathways. For each set, the boxes represent the first and third quartiles; yellow and black lines represent mean and median values, respectively. Significance of the reported difference on median values has been validated using the Mann–Whitney *U* test obtaining *p*-value = 0.04. # Number of.

In Figure 4, we show that on average EC proteins that are present in at least 10 KEGG metabolic pathways, and have the highest number of interacting partners, are also endowed with the highest number of interacting sites in the solvent accessible area. This finding supports the notion that the association of experimental and theoretical data is consistent and makes it feasible to identify possible hubs in metabolic pathways.

For the human EC proteins that most frequently (≥10 times) participate in KEGG metabolic pathways, Table 3 lists details including the most representative PDB structure (highest coverage to the protein sequence (≥70%) and highest atomic resolution). For each EC protein, we also indicate the putative number of predicted interaction sites (computed with ISPRED [24,25]), with the distinction among interaction sites at the protein solvent accessible surface or at the interface in the protein global stoichiometry, as reported in the PDB. We also show, for each EC protein, the total number of disease related variations and the number of disease related variations matching interactions sites.

### 3.3. The Case Study of Alpha-Aminoadipic Semialdehyde Dehydrogenase

The human protein alpha-aminoadipic semialdehyde (AASA) dehydrogenase, also known as antiquitin (P49419), coded by the gene ALDH7A1, is a multifunctional enzyme mediating important protective effects. The protein protects cells from oxidative stress by metabolizing lipid peroxidation-derived aldehydes (EC 1.2.1.3), and it is involved in lysine catabolism (EC 1.2.1.31). It also metabolizes betaine aldehyde to betaine (EC 1.2.1.8), an important cellular osmolyte and methyl donor. It is present with three different isoforms, one of which is only mitochondrial [19]. In human phenotype ontology [29], as reported in GeneCards, [19], the gene is associated to 59 human phenotypes and eight different REACTOME [5] and 13 KEGG [4] metabolic pathways (Table 3). In Gene Cards, expression data suggest that the protein is present in many tissues. GeneORGANizer [30] lists brain, cranial nerve, eye, head, liver, lung, peripheral nervous system, and peripheral nerve as confident expression organs. In the Human Protein Atlas [31], ALDH7A1 is associated with 34 reactions in 17 different subsystems—cytosol, endoplasmic reticulum, lysosome, mitochondria, and peroxisome. Given its relevance for the biology of the cell, it has been the subject of more than 100 publications (they can be reached via GeneCards [19]). The protein is present in the cytoplasm, in the mitochondrion, and in the nucleus [18] and interacts with other proteins (23 interactors in IntAct [14] and 62 in BioGRID [15]). It has been crystallized 15 times [7]. Here we focus on a complete form of the biological unit (PDB code: 4ZUL), a homotetramer solved with a resolution of 0.170 nm and with the maximal coverage with the sequence P49419, without the mitochondrial target peptide [32]. Recently, important variants of the protein, associated with PDE and hampering its activity, have been also solved with atomic resolution [33]. Finally, the protein, as a major feature, according to the MobiDB database [34], does not have intrinsically disordered regions (IDPs). We are interested in highlighting at a molecular level some of the protein properties, which are related to its involvement in different metabolic pathways and diseases.

A whole list of all the variations available from different databases is listed in Table S2. The protein sequence P49419 (comprising 539 residue) is endowed with 232 variations from different data bases (Table S2); 195 variations associated to 160 positions are disease related (Table S2), and 117 disease related variations are associated to PDE.

**Figure 5:**
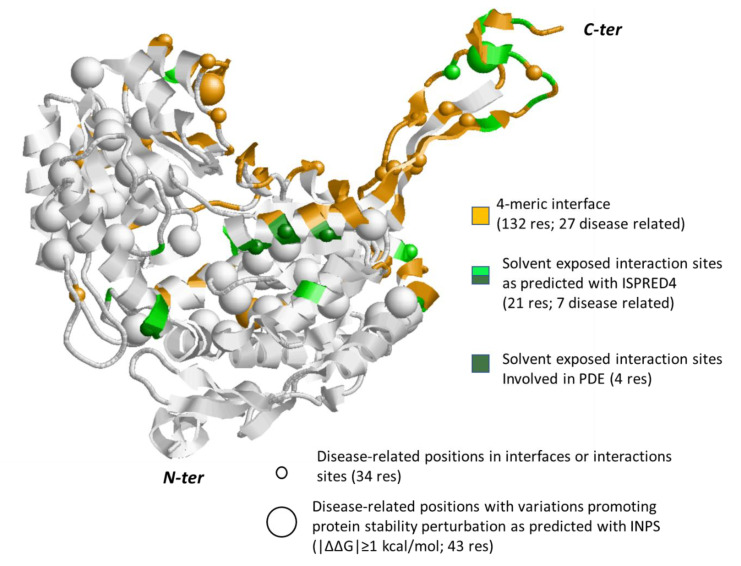
Monomeric subunit of human ALDH7A1 protein (PDB code: 4ZUL.A). Interaction surface in the tetramer as derived from the crystallographic coordinates is in orange. Interaction sites out the tetrameric interface, as predicted with ISPRED4, are in green. Positions in these regions carrying disease related variations (Table S2) are highlighted with small spheres. Big spheres highlight positions in the protein carrying disease related variations (Table S2, for details) and promoting a large variance of folding free energy, as predicted with INPS [26]. Grey color: the background protein backbone.

In Figure 5, we show one of the four subunits of the homotetrameric protein (4ZUL, chain A) and highlight the interface region (in orange) in the global stoichiometric unit. This allows distinguishing between the region at the interface and the region exposed to the solvent. We map (in green) variations predicted as possible interaction sites with ISPRED4 [24]. These sites, located in the protein-exposed region, are likely to mediate interactions with other proteins. We also map disease related residues at the interface and in the protein (small spheres). 

For the sake of completeness, we computed the likelihood of all the protein variations to affect protein stability (Table S2) and found as expected [2] that variations that are not always disease-related are perturbing the protein folding.

In Figure 5, big spheres highlight those variations that most affect protein stability (|∆∆G|≥1 kcal/mol). Interestingly, we found that PDE related variations V278L, Q281H, M285V, and K375R occur at the solvent accessible protein surface and match predicted interaction sites without affecting protein stability.

Table S2 provides a complete list of the properties for all the protein variations present in different databases, associated with specific diseases. For each variation, Table S2 lists its location in the protein reference sequence P49419, its location in the protein three-dimensional structure (4ZUL, chain A) and the predicted effect on the protein stability (∆∆G), computed with INPS, [26]. It also indicates if the disease-associated residue occurs in the target peptide, in the tetrameric interface, in the active sites, and regions annotated in the corresponding UniProt file (P49419). The ISPRED predictions are shown when present. Interestingly, many variations occur in the transit peptide (26 residue long, UniProt, P49419, [8]), a specific N-terminal peptide in the protein sequence mediating the mitochondrial import. This suggests that disease may be also due to an unpaired translocation of the protein to the mitochondrial compartment. For the sake of comparison, in Table S2 (Supplementary Materials), we label, in red, some PDE disease-related variations, known to occur in the aldehyde substrate binding site (N195S, P197S, A199V, G202V, W203G) and recently detailed with atomic resolution on their effect on the protein structure and function [33]. INPS predicts P197S, G202V, W203G as perturbing the protein stability (Table S2).

### 3.4. The Case Study of Acetyl-CoA C-Acetyltransferase

In Table 3, the enzyme proteins listed for the activity EC 2.3.1.9 are Acetyl-CoA C-acetyltransferases (ACAT2, cytosolic and ACAT1 mitochondrial), which catalyze the condensation of an acetyl-CoA and an acyl-CoA (often another acetyl-CoA), leading to the synthesis of an acyl-CoA with a longer fatty acid chain [35,36]. The two enzymes are encoded by two different genes and their residue chains share 39% sequence identity. The cytosolic enzyme (UniProt Q9BWD1) is encoded by ACAT2 and the mitochondrial one by ACAT1 (UniProt P24752). The 3D structure of both proteins has been resolved at the atomic resolution. Two representative structures (2IBY:A and 1WL4:A for ACAT1 and ACAT2, respectively) structurally superimpose with a root mean square deviation as low as 0.09 nm and therefore show a high structural similarity. Moreover, they conserve the two cysteine residues that form the active site.

In humans, ACAT1 is one of the enzymes that catalyzes the last step of the mitochondrial beta-oxidation pathway, an aerobic process breaking down fatty acids into acetyl-CoA, and it plays a major role in the metabolism of ketone bodies. ACAT2 is important in the pathway of fatty acid metabolism, and in the biosynthetic pathway of cholesterol. ACAT1 and ACAT2 are both associated with the same disease—alpha-methylacetoacetic aciduria (OMIM 203,750) or deficiency of acetyl-CoA acetyltransferase, an inborn error of isoleucine catabolism. They share 13 metabolic pathways (Table 3).

In human phenotype ontology [29], as reported in GeneCards, [19], ACAT1 is associated with 118 human phenotypes, while ACAT2 is associated with 23 human phenotypes.

GeneORGANizer [30] reports that brain and head are confident expression organs for both ACAT1 and ACAT2. ACAT1 is also expressed in liver, oesophagus, and stomach. In the Human Protein Atlas [31], ACAT1 is associated with two reactions in cytosol, mitochondria, and peroxisome. Given its relevance for the biology of the cell, the two proteins are subject of many publications that can be reached via GeneCards [19]. ACAT1 protein has 32 interactors in IntAct [14] and 108 in BioGRID [15], while ACAT2 has 20 interactors in IntAct [14] and 46 in BioGRID [15] (Table 3). Particularly for ACAT1, these numbers are significantly larger than the number of interactions per protein in the whole dataset. Finally, according to the MobiDB database [34], none of the proteins have intrinsically disordered regions (IDPs).

First, we focus on a complete form of the ACAT1 biological unit (PDB code: 2IBY), a homotetramer solved with a resolution of 0.185 nm. The monomeric chain covers all the mature form of P24752, depleted of the target peptide [35]. We are interested in highlighting at a molecular level some of the protein properties, which are related to its involvement in different metabolic pathways and diseases. A whole list of all the variations available from different databases is reported in Table S3. 

**Figure 6:**
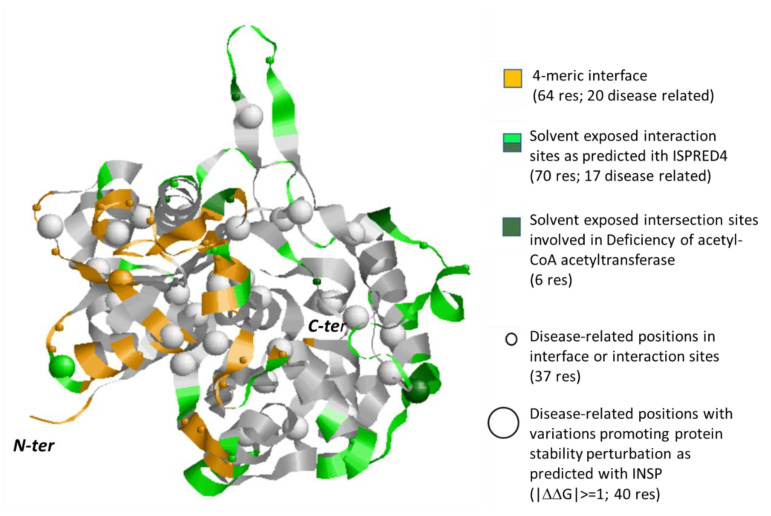
Monomeric subunit of human ACAT1 protein (PDB code: 2IBY.A). Interaction surface in the tetramer as derived from the crystallographic coordinates is in orange. Interaction sites out the tetrameric interface, as predicted with ISPRED4 are in green. Positions in these regions carrying disease related variations (Table S3) are highlighted with small spheres. Big spheres highlight positions in the protein carrying disease related variations (Table S3, for details) and promoting a large variance of folding free energy, as predicted with INPS [26]. Grey color: the background protein backbone.

In Figure 6, we show the subunit A of the homotetrameric protein, and we color the interface region in the global stoichiometric unit in orange and the residues predicted with ISPRED4 as possible interaction sites in green. As in Figure 5, we represent disease-related residues at the interface and in the protein with small spheres, while big spheres highlight variations that most affect protein stability (|∆∆G|≥1 kcal/mol). Table S3 provides a complete list of the properties for all the protein variations present in different databases, associated with specific diseases and mapped on the protein reference sequence (P24752) and three-dimensional structure (2IBY, chain A) on the protein.

As in the case of ALDH71, we found that variations in putative interaction sites are often conducive to the impairment of protein function. This is the case of eight variations related to Deficiency of acetyl-CoA acetyltransferase (Q73P, N158S, N158D, R208Q, R208G, T241A, R258C, T285I). Interestingly, five variations occur in the 33 residue-long mitochondrial target peptides, suggesting that disease may be also due to an unpaired translocation of the protein to the mitochondrial compartment.

For the ACAT2 protein, we adopted the PDB entry 1WL4 to represent the interaction regions and map the variations. The entry contains a homotetrameric form solved with a resolution of 0.155 nm. Each chain covers the whole sequence (Q9BWD1) [36].

**Figure 7:**
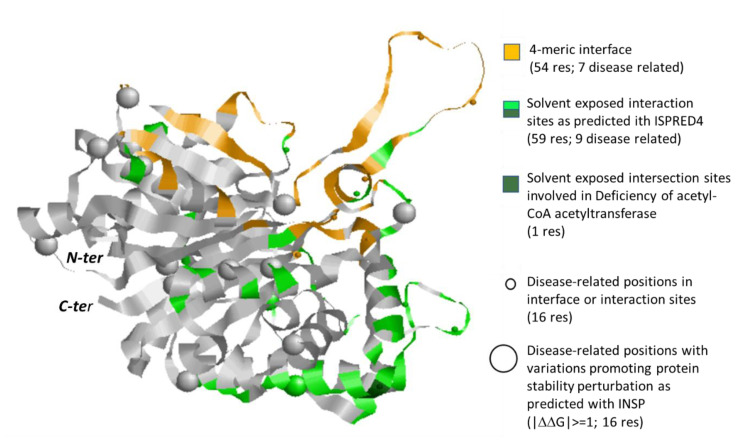
Monomeric subunit of human ACAT2 protein (PDB code: 1WL4.A). Interaction surface in the tetramer as derived from the crystallographic coordinates is in orange. Interaction sites out the tetrameric interface, as predicted with ISPRED4 are in green. Positions in these regions carrying disease related variations (Table S4 (Supplementary Materials)) are highlighted with small spheres. Big spheres highlight positions in the protein carrying disease related variations (Table S4, for details) and promoting a large variance of folding free energy, as predicted with INPS [26]. Grey color: the background protein backbone.

In Figure 7, we show the ACAT2 subunit chain A and represent tetrameric interaction regions, predicted interaction residues and positions carrying disease related variations with the same representation as in Figure 5 and Figure 6. Table S4 (Supplementary Materials) provides a complete list of the properties for all the protein variations present in different databases. Reinforcing the previous observations on the relevance of interaction regions, the only reported variation of ACAT2 associated with the Deficiency of acetyl-CoA acetyltransferase (E176K) occurs at the solvent accessible protein surface and it is predicted with ISPRED4 as interaction site. Moreover, this variation has a small effect on the protein stability (∆∆G) (−0.12 kcal/mol, see Table S4), reinforcing the concept that variations which are interaction sites can lead to disease by hampering protein-protein interactions without affecting protein stability.

## 4. Conclusions

One of the goals of system biology is to produce a three-dimensional model of the cell metabolism. As a preliminary step, nowadays, we cope with the problem of generating links among different databases that are dissecting the cell complexity into useful and important sets of data, addressing cell components from different perspectives and with different approaches. Here, we explore the problem of relating KEGG metabolic pathways to the network of protein–protein interactions (PPI) by restricting our study to human enzymes and their relation to KEGG metabolic pathways and PPI interaction maps. We found that, when enzymes are hubs in metabolic pathways, they are on average interacting with a high number of proteins as detected with different experimental methods and are also endowed with a high number of predicted interacting sites (Figure 3 and Figure 4).

Our results suggest that enzymatic metabolic hubs are hubs in networks of protein–protein interaction. Consistently, hubs are on average endowed with the highest numbers of predicted interaction sites when compared to the other EC proteins in the networks.

Protein variants can be associated with diseases. Possible indications on the effect of disease-related variations are investigated by predicting whether the variation is located at a putative interaction site and/or whether it affects the protein stability. As a test case, we focused on the ALDH7A1 gene, which according to our data is one of the most frequent gene in KEGG metabolic pathways. The protein is associated with 232 variations in different databases (Table S2 (Supplementary Materials)). We localize the disease-related variations in the protein structure and find that 27% of them affect the protein stability, rather independently of their location in active sites, in interfaces of the biological assembly or in the protein solvent exposed area (Table S2). The protein also interacts physically with 23–62 different interactors as documented in Intact and BioGrid (Table 3). We predict that 21 residues are likely to act as interaction sites in the solvent exposed protein surface (Table S2). Among these, seven are disease-related, and four are associated with PDE. This suggests that each disease-related variation occurring in the external surface can affect the efficiency of the protein in each of the different metabolic pathways where it is active, by affecting the interplay with all the different partners and without affecting protein stability. Similar conclusions stand also for the analysis of ACAT1 and ACAT2 gene products, representative of the second EC number of the list shown in Table 3. Again, by entering into the details of the molecular properties, we find a supportive example of the relevance of variations at the protein solvent accessible interface as conducive to disorders.

Summing up, a conclusion from our analysis is that, with the data presently available and with computational tools, it is possible to highlight enzyme proteins that are central to biochemical pathways and to identify possible molecular mechanisms at the basis of their association with specific diseases.

## Figures and Tables

**Table 1:** Disease-related human proteins with enzyme commission (EC) number.

Set	# Human Proteins
In SwissProt/UniProt	20365
Proteins with four-digit EC (EC proteins)	3428 (1411 EC) *
Proteins associated with genetic diseases	7316 (5788 diseases)
EC proteins with genetic disease associations	1669 (955 EC and 1900 diseases)

* Number of four-digit EC numbers.

**Table 2:** EC human protein in Kyoto Encyclopaedia of Genes and Genomes (KEGG) pathways.

Set	Human Proteins with KEGG Pathways		Human Proteins with KEGG Metabolic Pathways	
	#Proteins	#Pathways	#Proteins	#Pathways
In SwissProt	6904	320	1642	90
EC proteins	2258	317	1375	90
Proteins associated to genetic diseases	3391	320	895	90
EC proteins associated to genetic diseases	1255	314	770	90

# Number of.

**Table 3:** EC proteins involved in at least 10 KEGG metabolic pathways, and physical and predicted number of interactions.

EC Number ^1^	KEGG ^2^	UniProt ^3^	PDB ^4^	IntAct ^5^	BioGRID ^6^	Int. Sites ^7^	Other EC ^8^
1.2.1.3	13: hsa00010 hsa00053 hsa00071 hsa00260 hsa00280 hsa00310 hsa00330 hsa00340 hsa00380 hsa00410 hsa00561 hsa00620 hsa01100	P49419 (13)	4ZUL (homo 4-mer)	23	62	78/235; 34/83 (21/132; 7/34)	1.2.1.8 (1) 1.2.1.31 (1)
		P49189 (12) -hsa00260	6QAP (homo 4-mer)	10	38	48/223; 0/2 (10/139; none)	1.2.1.19 (0) 1.2.1.47 (1)
		P05091 (12) -hsa00260	1O02 (homo 4-mer)	45	75	88/223; 16/41 (19/133; 4/25)	−
		P51648 (12) -hsa00260	4QGK (homo 2-mer)	91	107	82/238; 19/54 (20/139; 4/32)	1.2.1.94 (0)
		P30837 (12)-hsa00260	-	41	93	111/517; 1/1	−
2.3.1.9	13: hsa00071 hsa00072 hsa00280 hsa00310 hsa00380 hsa00620 hsa00630 hsa00640 hsa00650 hsa00900 hsa01100 hsa01200 hsa01212	Q9BWD1 (13)	1WL4 (homo 4-mer)	20	46	94/175; 20/37 (66/113; 9/16)	−
		P24752 (13)	2IBY (homo 4-mer)	32	108	117/185; 35/59 (65/121;17/37)	−
2.6.1.1	11: hsa00220 hsa00250 hsa00270 hsa00330 hsa00350 hsa00360 hsa00400 hsa01100 hsa01200 hsa01210 hsa01230	P00505 (11)	5AX8 (homo 2-mer)	37	42	38/192; 5/32 (4/126; 0/21)	2.6.1.7 (0)
		P17174 (11)	6DND (1-mer)	12	73	46/200; 10/42	2.6.1.3 (0)
4.2.1.17	11: hsa00062 hsa00071 hsa00280 hsa00310 hsa00380 hsa00410 hsa00640 hsa00650 hsa01100 hsa01200 hsa01212	P40939 (11)	6DV2 (hetero 4-mer)	116	254	24/375; 6/65 (23/337; 5/57)	1.1.1.211 (2)
		P30084 (11)	2HW5 (homo 6-mer)	65	112	45/163; 9/37 (2/68; 2/16)	−
		Q08426 (10) - hsa00062	−	123	109	234/723; 53/150	5.3.3.8 (1) 1.1.1.35 (8)

Hyphens in table cells refer to lack of information. ^1^ The list of functional activity names corresponding to EC numbers is available in Table S1A. ^2^ Number of metabolic KEGG associated to the EC number and list of corresponding IDs; the list of names of KEGG pathways is available in Table S1B. ^3^ Human protein codes included in UniProt (SwissProt section). Among brackets, number of KEGG pathways listed in the second column where the protein is active. ^4^ Representative PDB code and corresponding global stoichiometry. ^5^ Number of interacting partners in IntAct. ^6^ Number of interacting partners in BioGRID. ^7^ Number of residues predicted with ISPRED to be involved in interactions with other proteins over the total number of residues on the protein solvent accessible surface. After the semicolon, we report the number of disease related positions matching the predicted interactions sites over the number of disease related positions on the protein solvent accessible surface. Within brackets, the same numbers are restricted to the residues not involved in the PDB global stoichiometry (biological unit). When structure is not available, the number of residues in the sequence is indicated instead of the number of surface residues. ^8^ Other EC numbers associated with the protein. Within brackets, the number of metabolic KEGG pathways, where the specific activity is present.

## Supplementary Materials

The following are available online at https://www.mdpi.com/2227-9059/8/8/250/s1.
