# Supplementary material for: Highlighting Human Enzymes Active in Different Metabolic Pathways and Diseases: The Case Study of EC 1.2.3.1 and EC 2.3.1.9
Source: Biomedicines. 2020 Jul 29;8(8):250. doi: 10.3390/biomedicines8080250 (PMC7459455; doi:10.3390/biomedicines8080250)
Supplement: Supplementary file 1 [file biomedicines-08-00250-s001.pdf]

biomedicines

Supplementary Table S1A. *List of EC numbers present in Table 3*

1.2.1.3	Aldehyde dehydrogenase (NAD(+))
2.3.1.9	Acetyl-CoA C-acetyltransferase
2.6.1.1	Aspartate transaminase
4.2.1.17	Enoyl-CoA hydratase

Supplementary Table S1B. *List of KEGG identifiers present in Table 3*

hsa00010	Glycolysis / Gluconeogenesis
hsa00053	Ascorbate and aldarate metabolism
hsa00062	Fatty acid elongation
hsa00071	Fatty acid degradation
hsa00072	Synthesis and degradation of ketone bodies
hsa00220	Arginine biosynthesis
hsa00250	Alanine, aspartate and glutamate metabolism
hsa00260	Glycine, serine and threonine metabolism
hsa00270	Cysteine and methionine metabolism
hsa00280	Valine, leucine and isoleucine degradation
hsa00310	Lysine degradation
hsa00330	Arginine and proline metabolism
hsa00340	Histidine metabolism
hsa00350	Tyrosine metabolism
hsa00360	Phenylalanine metabolism
hsa00380	Tryptophan metabolism
hsa00400	Phenylalanine, tyrosine and tryptophan biosynthesis
hsa00410	beta-Alanine metabolism
hsa00561	Glycerolipid metabolism
hsa00620	Pyruvate metabolism
hsa00630	Glyoxylate and dicarboxylate metabolism
hsa00640	Propanoate metabolism
hsa00650	Butanoate metabolism
hsa00900	Terpenoid backbone biosynthesis
hsa01100	Metabolic pathways
hsa01200	Carbon metabolism
hsa01210	2-Oxocarboxylic acid metabolism
hsa01212	Fatty acid metabolism
hsa01230	Biosynthesis of amino acids

biomedicines

MDPI

Supplementary Table S2: *Variants in ALDH7A1*

Position in UniProt	Position in PDB	Variant	dbSNP	Disease | Source	ΔΔG	RSA	surface	4-meric interface	Non 4-meric interface (predicted)	Sites/regions
3	-	R3L	rs759866910	DOID:1324 / lung cancer | Biomuta; Pyridoxine-dependent epilepsy | ClinVar; not provided | ClinVar	-	-	-			Target peptide
3	-	R3H	rs759866910	Pyridoxine-dependent epilepsy | ClinVar; not provided | ClinVar	-	-	-			Target peptide
3	-	R3P	rs759866910	not provided | ClinVar	-	-	-			Target peptide
3	-	R3C	rs796052265	not provided | ClinVar	-	-	-			Target peptide
5	-	P5L		Pyridoxine-dependent epilepsy | ClinVar	-	-	-			Target peptide
5	-	P5S	rs745921838	not provided | ClinVar	-	-	-			Target peptide
6	-	R6C	rs781779462	Pyridoxine-dependent epilepsy | ClinVar	-	-	-			Target peptide
7	-	A7V	rs1561674305	Pyridoxine-dependent epilepsy | ClinVar	-	-	-			Target peptide
8	-	L8P	rs796052255	not specified | ClinVar	-	-	-			Target peptide
11	-	H11E	rs796052269	Pyridoxine-dependent epilepsy | ClinVar; not specified | ClinVar	-	-	-			Target peptide
12	-	A12D		DOID:8618 / oral cavity cancer | Biomuta	-	-	-			Target peptide
18	-	L18P		Pyridoxine-dependent epilepsy | ClinVar	-	-	-			Target peptide

Position in UniProt	Position in PDB	Variant	dbSNP	Disease∣Source	ΔΔG	RSA	surface	4-meric interface	Non 4-meric interface (predicted)	Sites/regions
19	-	S19F	rs566243475	Pyridoxine-dependent epilepsy∣ClinVar; Seizures∣ClinVar; not provided∣ClinVar	-	-	-			Target peptide
22	-	W22C		Pyridoxine-dependent epilepsy∣ClinVar	-	-	-			Target peptide
23	-	S23G	rs796052266	not provided∣ClinVar	-	-	-			Target peptide
25	-	P25S	rs762624752	not provided∣ClinVar	-	-	-			Target peptide
27	-	A27P	rs1060502950	Pyridoxine-dependent epilepsy∣ClinVar	-	-	-			
29	-	M29V		Pyridoxine-dependent epilepsy∣ClinVar	-	-	-			
30	-	S30Y		DOID:0070003 / blastoma∣Biomuta; DOID:3571 / liver cancer∣Biomuta	-	-	-			
31	3	T31I		DOID:263 / kidney cancer∣Biomuta; DOID:9256 / colorectal cancer∣Biomuta	0.69	0.76	yes			
36	8	Q36H	rs779736481	not provided∣ClinVar	-0.31	0.28	yes			
38	10	Q38K		DOID:1324 / lung cancer∣Biomuta	-0.06	0.67	yes			
40	12	A40G	rs745385277	Pyridoxine-dependent epilepsy∣ClinVar	-0.63	0.49	yes			
40	12	A40V	rs745385277	Pyridoxine-dependent epilepsy∣ClinVar	0.30	0.49	yes			
47	19	L47V		DOID:363 / uterine cancer∣Biomuta	-0.57	0.02	no			
47	19	L47F		Pyridoxine-dependent epilepsy∣ClinVar	-0.37	0.02	no			
52	24	E52Q	rs765562932	Pyridoxine-dependent epilepsy∣ClinVar; Seizures∣ClinVar	-0.26	0.33	yes			
64	36	E64D		Pyridoxine-dependent epilepsy∣ClinVar	-0.42	0.37	yes			
67	39	T67M	rs543181020	Pyridoxine-dependent epilepsy∣ClinVar; not provided∣ClinVar	-0.45	0.27	yes			
68	40	T68N	rs58528748	Pyridoxine-dependent epilepsy∣ClinVar; Intractable seizure∣ClinVar; not provided∣ClinVar	-0.87	0.03	no			

Position in UniProt	Position in PDB	Variant	dbSNP	Disease∣Source	ΔΔG	RSA	surface	4-meric interface	Non 4-meric interface (predicted)	Sites/regions
69	41	Y69C	rs796052258	not provided∣ClinVar	-1.08	0.54	yes			
79	51	R79K	rs781361567	not provided∣ClinVar	-0.62	0.33	yes			
79	51	R79G	rs794727259	not provided∣ClinVar	-1.42	0.33	yes			
81	53	R81L		Pyridoxine-dependent epilepsy∣ClinVar	-0.30	0.27	yes			
82	54	Q82K		DOID:3571 / liver cancer∣Biomuta	-0.89	0.14	no			
83	55	A83T	rs1561672611	Pyridoxine-dependent epilepsy∣ClinVar	-0.81	0.06	no			
87	59	D87H		Pyridoxine-dependent epilepsy∣ClinVar; DOID:11934 / head and neck cancer∣Biomuta	-0.76	0.08	no			
88	60	Y88F	rs376294954	Pyridoxine-dependent epilepsy∣ClinVar	-0.72	0.02	no			
95	67	A95T	rs551163406	Pyridoxine-dependent epilepsy∣ClinVar	-1.47	0	no			
96	68	R96G		Pyridoxine-dependent epilepsy∣ClinVar	-1.17	0.37	yes			
98	70	A98S		DOID:1324 / lung cancer∣Biomuta	-1.15	0.06	no			
106	78	P106S		DOID:1909 / melanoma∣Biomuta	-0.48	0.61	yes		yes	
106	78	P106T		DOID:3571 / liver cancer∣Biomuta	-0.46	0.61	yes	yes	yes	
108	80	P108S		Pyridoxine-dependent epilepsy∣ClinVar; DOID:1909 / melanoma∣Biomuta	-0.47	0.3	yes	yes	yes	
109	81	K109T		DOID:1324 / lung cancer∣Biomuta	-0.31	0.65	yes	yes	yes	
109	81	K109Q		DOID:1324 / lung cancer∣Biomuta	0.19	0.65	yes	yes	yes	
110	82	R110L		DOID:10283 / prostate cancer∣Biomuta	0.32	0.06	no			
110	82	R110G	rs121912708	not specified∣ClinVar	-1.05	0.06	no			
111	83	G111E		Pyridoxine-dependent epilepsy∣ClinVar	-0.41	0	no			
114	86	V114A		DOID:1909 / melanoma∣Biomuta	-2.17	0	no			
119	91	D119N	rs1212969829	Pyridoxine-dependent epilepsy∣ClinVar; DOID:363 / uterine cancer∣Biomuta	-0.46	0.45	yes	yes		
120	92	A120D		Pyridoxine-dependent epilepsy∣ClinVar	-1.48	0.24	yes			

Position in UniProt	Position in PDB	Variant	dbSNP	Disease∣Source	ΔΔG	RSA	surface	4-meric interface	Non 4-meric interface (predicted)	Sites/regions
120	92	A120G	rs549279821	Pyridoxine-dependent epilepsy∣ClinVar	-1.35	0.24	yes			
122	94	R122P	rs796052256	Pyridoxine-dependent epilepsy∣ClinVar; not provided∣ClinVar	-1.26	0.38	yes	yes		
122	94	R122Q		Pyridoxine-dependent epilepsy∣ClinVar	-0.58	0.38	yes	yes		
122	94	R122W	rs370624118	Pyridoxine-dependent epilepsy∣ClinVar; DOID:3963 / thyroid carcinoma∣Biomuta; not specified∣ClinVar; not provided∣ClinVar; Seizures∣ClinVar; DOID:363 / uterine cancer∣Biomuta	-0.50	0.38	yes	yes		
125	97	I125V	rs117295656	Pyridoxine-dependent epilepsy∣ClinVar; not specified∣ClinVar; Seizures∣ClinVar; not provided∣ClinVar	-0.29	0.15	no			
129	101	G129V		DOID:363 / uterine cancer∣Biomuta	2.04	0	no			
144	116	V144M		Pyridoxine-dependent epilepsy∣ClinVar	0.58	0.18	no			
148	120	Q148K		DOID:3571 / liver cancer∣Biomuta	-0.32	0.38	yes	yes		
151	123	V151A	rs1561667040	Seizures∣ClinVar	-1.13	0	no			
152	124	D152H		DOID:11054 / urinary bladder cancer∣Biomuta	-0.16	0.36	yes	yes	yes	
154	126	C154R	rs1057520885	not provided∣ClinVar	-1.90	0	no			
157	129	A157S		DOID:0070003 / blastoma∣Biomuta	-1.05	0	no			
160	132	L160V		DOID:1324 / lung cancer∣Biomuta	-0.94	0.09	no			
165	137	G165E		DOID:11934 / head and neck cancer∣Biomuta	0.11	0.43	yes	yes		
165	137	G165V		Pyridoxine-dependent epilepsy∣ClinVar	0.05	0.43	yes	yes		
165	137	G165A	rs375491094	not provided∣ClinVar	0.02	0.43	yes	yes		
168	140	I168T		DOID:363 / uterine cancer∣Biomuta	-0.26	0.84	yes	yes	yes	
171	143	S171F		DOID:363 / uterine cancer∣Biomuta	-0.25	0.23	yes		yes	
173	145	R173T		DOID:1324 / lung cancer∣Biomuta	-1.06	0.28	yes		yes	

Position in UniProt	Position in PDB	Variant	dbSNP	Disease	Source	ΔΔG	RSA	surface	4-meric interface	Non 4-meric interface (predicted)	Sites/regions
177	149	A177P	rs140102105	not provided | ClinVar	-0.86	0.2	no			
177	149	A177E	rs764417585	Pyridoxine-dependent epilepsy | ClinVar; EPILEPSY. PYRIDOXINE-DEPENDENT | DisGeNet	-0.39	0.2	no			
178	150	L178M		DOID:3571 / liver cancer | Biomuta	-0.26	0.24	yes	yes	yes	
179	151	I179T	rs763217060	not provided | ClinVar	-1.19	0.2	no			
181	153	Q181R	rs796052257	Pyridoxine-dependent epilepsy | ClinVar; not provided | ClinVar	0.37	0.45	yes	yes	yes	
185	157	V185L	rs61757685	Pyridoxine-dependent epilepsy | ClinVar	0.01	0.14	no			
185	157	V185I	rs61757685	Pyridoxine-dependent epilepsy | ClinVar; not provided | ClinVar	-0.04	0.14	no			
190	162	I190F		DOID:1324 / lung cancer | Biomuta	-1.59	0	no			
191	163	I191V	rs1060499755	Abnormality of brain morphology | DisGeNet; Abnormality of brain morphology | ClinVar	-0.97	0.05	no			
192	164	T192M	rs376917645	Pyridoxine-dependent epilepsy | ClinVar; not provided | ClinVar	-0.52	0.04	no			
193	165	A193T	rs773503933	DOID:0070003 / blastoma | Biomuta; Pyridoxine-dependent epilepsy | ClinVar; DOID:3571 / liver cancer | Biomuta; not provided | ClinVar	-0.70	0.18	no			
195	167	N195S	rs372660425	Pyridoxine-dependent epilepsy | ClinVar; EPILEPSY. PYRIDOXINE-DEPENDENT | DisGeNet; not provided | ClinVar	-0.71	0.04	no			Transition state stabilizer
196	168	F196L		DOID:9256 / colorectal cancer | Biomuta	-2.07	0.12	no			
197	169	P197L		DOID:1909 / melanoma | Biomuta	-0.47	0	no			

Position in UniProt	Position in PDB	Variant	dbSNP	Disease∣Source	ΔΔG	RSA	surface	4-meric interface	Non 4-meric interface (predicted)	Sites/regions
197	169	P197S	rs779652673	Pyridoxine-dependent epilepsy∣ClinVar; EPILEPSY. PYRIDOXINE-DEPENDENT∣DisGeNet; not provided∣ClinVar	-1.21	0	no			
199	171	A199V	rs121912709	Pyridoxine-dependent epilepsy∣ClinVar; Pyridoxine-dependent epilepsy (PDE)∣HUMSAVAR; EPILEPSY. PYRIDOXINE-DEPENDENT∣DisGeNet	-0.28	0	no			
200	172	V200A		DOID:363 / uterine cancer∣Biomuta	-1.71	0.06	no			
202	174	G202V		DOID:2531 / hematologic cancer∣Biomuta; Pyridoxine-dependent epilepsy (PDE)∣HUMSAVAR	1.92	0	no			
203	175	W203G	rs555896752	not provided∣ClinVar	-3.13	0.06	no			
208	180	A208V	rs200433565	Seizures∣ClinVar	-1.38	0	no			
211	183	C211F	rs1131691976	not provided∣ClinVar	-1.76	0	no			
216	188	L216P		Pyridoxine-dependent epilepsy∣ClinVar	-3.14	0	no			
219	191	G219R		DOID:3963 / thyroid carcinoma∣Biomuta	-0.65	0.02	no			
219	191	G219A		Pyridoxine-dependent epilepsy∣ClinVar	-0.01	0.02	no			
219	191	G219E		DOID:1324 / lung cancer∣Biomuta	-1.08	0.02	no			
222	194	T222A	rs777829351	Pyridoxine-dependent epilepsy∣ClinVar; not provided∣ClinVar	-0.47	0.4	yes			
223	195	T223N		DOID:3571 / liver cancer∣Biomuta	-1.08	0	no			
224	196	S224F		Pyridoxine-dependent epilepsy∣ClinVar	0.05	0.02	no			
225	197	L225F		DOID:9256 / colorectal cancer∣Biomuta	-0.90	0	no			
228	200	V228M	rs201832111	Pyridoxine-dependent epilepsy∣ClinVar	-0.24	0	no			
228	200	V228A		Pyridoxine-dependent epilepsy∣ClinVar	-0.54	0	no			
229	201	A229V	rs1473102872	Pyridoxine-dependent epilepsy∣ClinVar	0.06	0	no			
237	209	V237F		DOID:1909 / melanoma∣Biomuta	-1.10	0.08	no			

Position in UniProt	Position in PDB	Variant	dbSNP	Disease∣Source	ΔΔG	RSA	surface	4-meric interface	Non 4-meric interface (predicted)	Sites/regions
260	232	A260T		DOID:10283 / prostate cancer∣Biomuta	-0.82	0.25	yes			
261	233	M261V		Pyridoxine-dependent epilepsy∣ClinVar	-0.93	0	no			
262	234	A262T		DOID:363 / uterine cancer∣Biomuta	-0.48	0	no			
263	235	K263R		Pyridoxine-dependent epilepsy∣ClinVar	0.13	0.6	yes			
266	238	R266Q	rs796052259	not provided∣ClinVar	-1.15	0.25	yes			
272	244	F272L		DOID:9256 / colorectal cancer∣Biomuta	-2.09	0.08	no			
276	248	T276P	rs796052260	not provided∣ClinVar	-1.11	0.2	no			Nucleotide binding
278	250	V278L		Pyridoxine-dependent epilepsy∣ClinVar	-0.85	0.52	yes		yes	Nucleotide binding
281	253	Q281H		Pyridoxine-dependent epilepsy∣ClinVar	0.13	0.46	yes		yes	
281	253	Q281K		DOID:3571 / liver cancer∣Biomuta	-0.05	0.46	yes		yes	
285	257	M285V		Pyridoxine-dependent epilepsy∣ClinVar	-0.83	0.24	yes		yes	
287	259	Q287R	rs796052261	Pyridoxine-dependent epilepsy∣ClinVar; not provided∣ClinVar	-0.04	0.61	yes	yes		
289	261	R289W		DOID:3571 / liver cancer∣Biomuta	-1.03	0.12	no			
291	263	G291W		Pyridoxine-dependent epilepsy∣ClinVar	-0.42	0.27	yes			
291	263	G291E		Pyridoxine-dependent epilepsy (PDE)∣HUMSAVAR	-0.61	0.27	yes			
296	268	E296K		DOID:1909 / melanoma∣Biomuta	-0.68	0.04	no			Proton acceptor
298	270	G298R		DOID:363 / uterine cancer∣Biomuta	-0.61	0.01	no			
300	272	N300D		Pyridoxine-dependent epilepsy∣ClinVar	-0.59	0.03	no			
301	273	N301I	rs121912711	Pyridoxine-dependent epilepsy∣ClinVar; Pyridoxine-dependent epilepsy (PDE)∣HUMSAVAR; EPILEPSY. PYRIDOXINE-DEPENDENT∣DisGeNet	-0.02	0.01	no			

Position in UniProt	Position in PDB	Variant	dbSNP	Disease∣Source	ΔΔG	RSA	surface	4-meric interface	Non 4-meric interface (predicted)	Sites/regions
303	275	I303V		DOID:1324 / lung cancer∣Biomuta; Pyridoxine-dependent epilepsy∣ClinVar	0.07	0.01	no			
305	277	A305S		DOID:1324 / lung cancer∣Biomuta; DOID:1909 / melanoma∣Biomuta	-0.80	0	no			
305	277	A305G	rs141775154	Pyridoxine-dependent epilepsy∣ClinVar; not provided∣ClinVar	-1.66	0	no			
312	284	S312R	rs1561654302	Pyridoxine-dependent epilepsy∣ClinVar	0.36	0.54	yes	yes		
316	288	P316S		DOID:1909 / melanoma∣Biomuta	-0.29	0.54	yes	yes		
318	290	A318T	rs936151635	Pyridoxine-dependent epilepsy∣ClinVar	-0.87	0.02	no			
318	290	A318P	rs936151635	Pyridoxine-dependent epilepsy∣ClinVar	-2.04	0.02	no			
319	291	L319H	rs1057522342	not provided∣ClinVar	-1.59	0.21	yes	yes	yes	
321	293	A321T	rs746212816	DOID:3070 / malignant glioma∣Biomuta; not provided∣ClinVar	-1.09	0.01	no			
327	299	G327S	rs1554099008	not provided∣ClinVar	-0.85	0	no			
329	301	R329G		DOID:3963 / thyroid carcinoma∣Biomuta	-1.89	0.1	no			
329	301	R329K	rs864622558	Pyridoxine-dependent epilepsy∣ClinVar; EPILEPSY. PYRIDOXINE-DEPENDENT∣DisGeNet	-1.25	0.1	no			
329	301	R329S	rs761295869	Pyridoxine-dependent epilepsy∣ClinVar	-1.23	0.1	no			
332	304	T332I	rs543182575	Pyridoxine-dependent epilepsy∣ClinVar	-0.89	0.01	no			
332	304	T332S	rs543182575	Pyridoxine-dependent epilepsy∣ClinVar; Seizures∣ClinVar	-0.06	0.01	no			

Position in UniProt	Position in PDB	Variant	dbSNP	Disease∣Source	ΔΔG	RSA	surface	4-meric interface	Non 4-meric interface (predicted)	Sites/regions
333	305	A333V	rs144625212	Pyridoxine-dependent epilepsy∣ClinVar; not specified∣ClinVar	0.09	0.03	no			
335	307	R335Q	rs754449549	Pyridoxine-dependent epilepsy∣ClinVar; Pyridoxine-dependent epilepsy (PDE)∣HUMSAVAR; EPILEPSY. PYRIDOXINE-DEPENDENT∣DisGeNet; not provided∣ClinVar	-1.12	0.01	no			
337	309	F337C	rs747834606	Pyridoxine-dependent epilepsy∣ClinVar	-2.35	0	no			
338	310	I338L		DOID:2531 / hematologic cancer∣Biomuta	-0.71	0	no			
339	311	H339R	rs199767457	Pyridoxine-dependent epilepsy∣ClinVar; not provided∣ClinVar	-0.78	0.16	no			
341	313	S341N	rs141701364	Pyridoxine-dependent epilepsy∣ClinVar; not specified∣ClinVar	-0.46	0.47	yes			
346	318	V346A		DOID:1909 / melanoma∣Biomuta	-2.06	0	no			
349	321	R349T	rs553114356	Pyridoxine-dependent epilepsy∣ClinVar; not provided∣ClinVar	-0.83	0.4	yes			
355	327	A355T		DOID:9256 / colorectal cancer∣Biomuta	-0.49	0.58	yes			
356	328	Q356E	rs138675705	Pyridoxine-dependent epilepsy∣ClinVar; not provided∣ClinVar	-0.04	0.78	yes			
356	328	Q356R		DOID:1909 / melanoma∣Biomuta	0.13	0.78	yes			
357	329	I357V	rs761011399	not specified∣ClinVar	-0.31	0.11	no			
358	330	R358L	rs144671885	Pyridoxine-dependent epilepsy∣ClinVar; Seizures∣ClinVar	0.18	0.45	yes	yes		
358	330	R358Q		DOID:1793 / pancreatic cancer∣Biomuta	-0.58	0.45	yes	yes		
362	334	P362S	rs532800318	Pyridoxine-dependent epilepsy∣ClinVar; not provided∣ClinVar	-1.10	0	no			
363	335	W363R	rs886044173	not provided∣ClinVar	-0.96	0.19	no			

Position in UniProt	Position in PDB	Variant	dbSNP	Disease∣Source	ΔΔG	RSA	surface	4-meric interface	Non 4-meric interface (predicted)	Sites/regions
364	336	D364N		DOID:10283 / prostate cancer∣Biomuta	-0.30	0.4	yes			
365	337	P365H		DOID:3571 / liver cancer∣Biomuta	-0.37	0.85	yes			
365	337	P365S	rs370869432	Pyridoxine-dependent epilepsy∣ClinVar; DOID:9256 / colorectal cancer∣Biomuta; not provided∣ClinVar	-0.46	0.85	yes			
366	338	N366K		Pyridoxine-dependent epilepsy∣ClinVar	-0.12	0.63	yes			
375	347	K375R		Pyridoxine-dependent epilepsy∣ClinVar	-0.56	0.28	yes		yes	
376	348	Q376K	rs564187364	Pyridoxine-dependent epilepsy∣ClinVar; not provided∣ClinVar	-0.41	0.32	yes			
377	349	A377T		Pyridoxine-dependent epilepsy∣ClinVar	-0.80	0.16	no			
378	350	V378L		DOID:1793 / pancreatic cancer∣Biomuta	-0.34	0.13	no			
380	352	M380I		DOID:3571 / liver cancer∣Biomuta	0.03	0.68	yes			
384	356	A384E		DOID:3571 / liver cancer∣Biomuta	-1.55	0.05	no			
384	356	A384T	rs140947675	Pyridoxine-dependent epilepsy∣ClinVar; Leukoencephalopathy∣ClinVar; not provided∣ClinVar	-0.78	0.05	no			
386	358	E386K		DOID:3371 / chondrosarcoma∣Biomuta	-0.91	0.2	no			
388	360	A388G	rs1561651205	Pyridoxine-dependent epilepsy∣ClinVar	-1.54	0	no			
394	366	T394I		DOID:1324 / lung cancer∣Biomuta	-0.25	0.58	yes			
395	367	V395G		Pyridoxine-dependent epilepsy (PDE)∣HUMSAVAR	-3.27	0.26	yes			
398	370	G398V	rs864622557	Pyridoxine-dependent epilepsy∣ClinVar; EPILEPSY. PYRIDOXINE-DEPENDENT∣DisGeNet	-1.06	0.18	no			
399	371	G399V		Pyridoxine-dependent epilepsy∣ClinVar	-0.83	0.13	no			
403	375	D403N	rs796052262	not provided∣ClinVar	0.04	1	yes			
404	376	R404C		DOID:1909 / melanoma∣Biomuta	-0.59	0.41	yes			

Position in UniProt	Position in PDB	Variant	dbSNP	Disease ǀ Source	ΔΔG	RSA	surface	4-meric interface	Non 4-meric interface (predicted)	Sites/regions
404	376	R404H		DOID:10534 / stomach cancer ǀ Biomuta; DOID:9256 / colorectal cancer ǀ Biomuta; DOID:1909 / melanoma ǀ Biomuta	-0.51	0.41	yes			
409	381	V409L	rs796052263	not provided ǀ ClinVar	-0.95	0	no			
411	383	P411L	rs780233639	Seizures ǀ ClinVar; not provided ǀ ClinVar	-0.62	0.04	no			
411	383	P411Q		DOID:0070003 / blastoma ǀ Biomuta; DOID:3571 / liver cancer ǀ Biomuta	-0.78	0.04	no			
412	384	T412I		Pyridoxine-dependent epilepsy ǀ ClinVar; DOID:3963 / thyroid carcinoma ǀ Biomuta	-0.54	0	no			
412	384	T412A	rs2306618	not specified ǀ ClinVar; Pyridoxine-dependent epilepsy ǀ ClinVar; Polymorphism ǀ HUMSAVAR; DOID:9256 / colorectal cancer ǀ Biomuta; Seizures ǀ ClinVar	-1.12	0	no			
414	386	V414M		DOID:1324 / lung cancer ǀ Biomuta; Pyridoxine-dependent epilepsy ǀ ClinVar	-1.14	0	no			
415	387	T415K		DOID:3070 / malignant glioma ǀ Biomuta	-1.19	0.14	no			
415	387	T415I		Pyridoxine-dependent epilepsy ǀ ClinVar	-0.17	0.14	no			
418	390	G418D		DOID:263 / kidney cancer ǀ Biomuta	0.33	0.48	yes			
421	393	A421V	rs753598402	not provided ǀ ClinVar	-0.78	0.15	no			
423	395	I423V	rs760266177	not provided ǀ ClinVar	-0.43	0.08	no			
424	396	A424T		Pyridoxine-dependent epilepsy ǀ ClinVar	-0.81	0	no			
426	398	T426A	rs150305320	Pyridoxine-dependent epilepsy ǀ ClinVar; not provided ǀ ClinVar	-0.81	0.49	yes			
427	399	E427G		DOID:4362 / cervical cancer ǀ Biomuta	-0.76	0.24	yes			
427	399	E427D	rs796052271	not provided ǀ ClinVar	-0.69	0.24	yes			

Position in UniProt	Position in PDB	Variant	dbSNP	Disease∣Source	ΔΔG	RSA	surface	4-meric interface	Non 4-meric interface (predicted)	Sites/regions
427	399	E427Q	rs121912707	Pyridoxine-dependent epilepsy∣ClinVar; Pyridoxine-dependent epilepsy (PDE)∣HUMSAVAR; EPILEPSY. PYRIDOXINE-DEPENDENT∣DisGeNet; not provided∣ClinVar; Seizures∣DisGeNet; Epilepsy∣DisGeNet; Seizures∣ClinVar	-0.55	0.24	yes			
430	402	A430S		DOID:1612 / breast cancer∣Biomuta	-0.49	0.01	no			
430	402	A430V		DOID:1612 / breast cancer∣Biomuta	-0.46	0.01	no			
431	403	P431A		DOID:1793 / pancreatic cancer∣Biomuta	-0.96	0.02	no			
433	405	L433P		DOID:9256 / colorectal cancer∣Biomuta	-3.50	0.01	no			
434	406	Y434S	rs747597620	not provided∣ClinVar	-1.80	0.02	no			
434	406	Y434C	rs747597620	DOID:2994 / germ cell cancer∣Biomuta; Pyridoxine-dependent epilepsy∣ClinVar; DOID:2394 / ovarian cancer∣Biomuta; not provided∣ClinVar	-0.86	0.02	no			
439	411	K439Q	rs12514417	not specified∣ClinVar; Pyridoxine-dependent epilepsy∣ClinVar; Polymorphism∣HUMSAVAR; DOID:9256 / colorectal cancer∣Biomuta; Seizures∣ClinVar	-1.05	0.21	yes			
441	413	E441K		DOID:11054 / urinary bladder cancer∣Biomuta	-0.97	0.18	no			
446	418	A446V		DOID:2394 / ovarian cancer∣Biomuta	-0.88	0.49	yes			
452	424	K452N	rs756859037	Pyridoxine-dependent epilepsy∣ClinVar	-0.72	0.32	yes			
458	430	S458N		Pyridoxine-dependent epilepsy (PDE)∣HUMSAVAR	-0.70	0	no			
459	431	I459F	rs186558364	not provided∣ClinVar	-1.82	0	no			

Position in UniProt	Position in PDB	Variant	dbSNP	Disease∣Source	ΔΔG	RSA	surface	4-meric interface	Non 4-meric interface (predicted)	Sites/regions
459	431	I459T	rs1057518529	Pyridoxine-dependent epilepsy∣ClinVar; not specified∣ClinVar	-2.99	0	no			
459	431	I459M		DOID:1324 / lung cancer∣Biomuta	-1.66	0	no			
463	435	D463Y		DOID:363 / uterine cancer∣Biomuta	-0.17	0.41	yes			
465	437	G465D		DOID:363 / uterine cancer∣Biomuta	0.08	0.33	yes	yes	yes	
469	441	R469C		DOID:3070 / malignant glioma∣Biomuta	-0.50	0.43	yes			
469	441	R469H	rs147940248	Pyridoxine-dependent epilepsy∣ClinVar; not provided∣ClinVar	-0.62	0.43	yes			
471	443	L471P		DOID:9256 / colorectal cancer∣Biomuta	-2.33	0.64	yes	yes	yes	
471	443	L471F		Pyridoxine-dependent epilepsy∣ClinVar; DOID:10534 / stomach cancer∣Biomuta	-0.60	0.64	yes	yes	yes	
474	446	K474E		DOID:10283 / prostate cancer∣Biomuta	0.05	0.86	yes	yes	yes	
477	449	D477E		DOID:3571 / liver cancer∣Biomuta	-0.35	0.3	yes	yes		
480	452	I480T	rs886059849	Pyridoxine-dependent epilepsy∣ClinVar	-0.99	0.12	no			
481	453	V481L	rs796052264	not provided∣ClinVar	-1.20	0.3	yes	yes	yes	
481	453	V481E	rs144701796	not provided∣ClinVar	-1.84	0.3	yes	yes	yes	
484	456	N484S	rs1561648688	Pyridoxine-dependent epilepsy∣ClinVar	-0.05	0.3	yes	yes		
487	459	T487A		DOID:2531 / hematologic cancer∣Biomuta	-1.14	0.01	no			
494	466	G494V	rs763418390	Pyridoxine-dependent epilepsy∣ClinVar; not provided∣ClinVar	0.79	0.44	yes	yes		
505	477	G505R	rs556400964	Pyridoxine-dependent epilepsy∣ClinVar; EPILEPSY. PYRIDOXINE-DEPENDENT∣DisGeNet; not provided∣ClinVar	-0.65	0.05	no			
507	479	E507A	rs80049945	Pyridoxine-dependent epilepsy∣ClinVar	-0.76	0	no			
511	483	D511G		Pyridoxine-dependent epilepsy∣ClinVar	-0.94	0.45	yes	yes		

Position in UniProt	Position in PDB	Variant	dbSNP	Disease∣Source	ΔΔG	RSA	surface	4-meric interface	Non 4-meric interface (predicted)	Sites/regions
516	488	Y516H		DOID:0070003 / blastoma∣Biomuta; DOID:3571 / liver cancer∣Biomuta	-1.28	0	no			
516	488	Y516C		Pyridoxine-dependent epilepsy∣ClinVar	-2.04	0	no			
517	489	M517I	rs1064794774	not provided∣ClinVar	-1.01	0.02	no			
519	491	R519M		DOID:363 / uterine cancer∣Biomuta	-0.21	0.54	yes	yes	yes	
519	491	R519K	rs561343926	Pyridoxine-dependent epilepsy∣ClinVar	-0.47	0.54	yes	yes	yes	
520	492	S520F	rs1554097854	Pyridoxine-dependent epilepsy∣ClinVar; DOID:9256 / colorectal cancer∣Biomuta	-0.07	0.24	yes	yes	yes	
522	494	C522S	rs1057521316	not provided∣ClinVar	-0.92	0.24	yes	yes	yes	
523	495	T523N		DOID:363 / uterine cancer∣Biomuta	-0.34	0.45	yes	yes	yes	
523	495	T523A	rs61757684	Pyridoxine-dependent epilepsy∣ClinVar; not specified∣ClinVar; Seizures∣ClinVar; not provided∣ClinVar	-0.26	0.45	yes	yes	yes	
524	496	I524V		DOID:263 / kidney cancer∣Biomuta	-0.39	0.36	yes	yes	yes	
530	502	L530F		DOID:0070003 / blastoma∣Biomuta; DOID:3571 / liver cancer∣Biomuta	-0.03	0.87	yes	yes	yes	

Variants in ALDH7A1 are derived from different sources including Humsavar, OMIM, ClinVar, Biomuta,. Variants are mapped on the PDB file 4ZUL, chain A. ΔΔG values are predicted with INPS starting from structure. RSA= Relative Solvent Accessibility computed with DSSP. Surface residues are those with RSA ≥ 0.2. Residues that are part of the tetrameric interface are computed from the Biological Assembly of 4ZUL. Residues in non-tetrameric interfaces are predicted with ISPRED4. Sites and regions are derived from UniProt annotations. In red we highlighted the mutation reported in [33].

Supplementary Table S3: *Variants in ACAT1*

Position in UniProt	Position in PDB	Variant	dbSNP	Disease∣Source	ΔΔG	RSA	surface	4-meric interface	Non 4-meric interface (predicted)	Sites/regions
1	-	M1K	rs120074142	Deficiency of acetyl-CoA acetyltransferase∣ClinVar						Target peptide
1	-	M1T	rs120074142	Deficiency of acetyl-CoA acetyltransferase∣ClinVar						Target peptide
1	-	M1V	rs1305448140	Deficiency of acetyl-CoA acetyltransferase∣ClinVar						Target peptide
5	-	A5P	rs3741056	Deficiency of acetyl-CoA acetyltransferase∣ClinVar; Polymorphism∣HUMSAVAR; not specified∣ClinVar; DOID:9256 / colorectal cancer∣Biomuta						Target peptide
10	-	S10N	rs886047595	Deficiency of acetyl-CoA acetyltransferase∣ClinVar						Target peptide
13	-	R13G		DOID:3571 / liver cancer∣Biomuta						Target peptide
27	-	R27I		DOID:363 / uterine cancer∣Biomuta						Target peptide
29	-	V29L	rs764142914	DOID:10283 / prostate cancer∣Biomuta; DOID:1793 / pancreatic cancer∣Biomuta; not provided∣ClinVar						Target peptide
31	31	R31W	rs199952982	not provided∣ClinVar						Target peptide
35	35	S35L		DOID:1612 / breast cancer∣Biomuta						
36	36	K36Q		DOID:1324 / lung cancer∣Biomuta						
45	45	V45L		DOID:363 / uterine cancer∣Biomuta	-1.17	0.06	no			
52	52	I52T		Deficiency of acetyl-CoA acetyltransferase∣ClinVar	-2.47	0	no			

Position in UniProt	Position in PDB	Variant	dbSNP	Disease∣Source	ΔΔG	RSA	surface	4-meric interface	Non 4-meric interface (predicted)	Sites/regions
55	55	F55C		DOID:363 / uterine cancer∣Biomuta	-1.95	0.39	yes		yes	
57	57	G57C		DOID:1324 / lung cancer∣Biomuta	0.01	0.19	no			
66	66	K66N		DOID:3571 / liver cancer∣Biomuta	-0.53	0.5	yes		yes	
68	68	G68V	rs794727475	not provided∣ClinVar	0.26	0	no			
73	73	Q73P	rs779758622	Deficiency of acetyl-CoA acetyltransferase∣ClinVar	-0.93	0.46	yes		yes	
75	75	A75V		DOID:363 / uterine cancer∣Biomuta	-1.03	0	no			
81	81	I81N		DOID:10283 / prostate cancer∣Biomuta	-3.19	0.09	no			
89	89	A89V		DOID:3371 / chondrosarcoma∣Biomuta	1.73	0	no			
90	90	Y90C		DOID:9256 / colorectal cancer∣Biomuta	-1.26	0.16	no	yes		
92	92	G92S	rs1591362402	Deficiency of acetyl-CoA acetyltransferase∣ClinVar	-0.72	0	no			
93	93	N93S	rs120074145	Deficiency of acetyl-CoA acetyltransferase∣ClinVar; Deficiency of acetyl-CoA acetyltransferase∣DisGeNet; 3-ketothiolase deficiency (3KTD) [MIM:203750]∣HUMSAVAR; Ketoacidosis∣DisGeNet	-0.49	0	no			
98	98	G98V		DOID:2394 / ovarian cancer∣Biomuta	-0.86	0.95	yes	yes	yes	
99	99	E99D		DOID:1324 / lung cancer∣Biomuta	-0.74	0.18	no			
100	100	G100E	rs1591362472	Deficiency of acetyl-CoA acetyltransferase∣ClinVar	-0.82	0.56	yes	yes	yes	
101	101	Q101K	rs1591362483	Deficiency of acetyl-CoA acetyltransferase∣ClinVar	-0.16	0.92	yes	yes	yes	
103	103	P103H		DOID:1909 / melanoma∣Biomuta	-1.07	0.07	no			
116	116	S116Y		DOID:363 / uterine cancer∣Biomuta	-0.02	0.72	yes	yes	no	
117	117	T117P		DOID:363 / uterine cancer∣Biomuta	-1.11	0.01	no			

Position in UniProt	Position in PDB	Variant	dbSNP	Disease∣Source	ΔΔG	RSA	surface	4-meric interface	Non 4-meric interface (predicted)	Sites/regions
118	118	P118A		DOID:1324 / lung cancer∣Biomuta; DOID:11054 / urinary bladder cancer∣Biomuta; DOID:10534 / stomach cancer∣Biomuta	-0.99	0.46	yes	yes	yes	
124	124	K124R	rs1037467160	Deficiency of acetyl-CoA acetyltransferase∣ClinVar	-0.15	0.2	yes	yes	yes	
124	124	K124E	rs1591363715	Deficiency of acetyl-CoA acetyltransferase∣ClinVar	-1.03	0.2	yes	yes	yes	
126	126	C126S	rs1278227329	Deficiency of acetyl-CoA acetyltransferase∣ClinVar	-					Active site
127	127	A127V	rs1591363760	Deficiency of acetyl-CoA acetyltransferase∣ClinVar	-1.27	0.03	no			
129	129	G129E		DOID:11054 / urinary bladder cancer∣Biomuta	-1.14	0	no			
130	130	M130I		DOID:2994 / germ cell cancer∣Biomuta; DOID:2394 / ovarian cancer∣Biomuta	-0.96	0	no			
132	132	A132G	rs1591363786	Deficiency of acetyl-CoA acetyltransferase∣ClinVar	-1.64	0	no			
134	134	M134I		DOID:1324 / lung cancer∣Biomuta	-0.49	0.01	no			
135	135	M135V		DOID:9256 / colorectal cancer∣Biomuta	-0.77	0.35	yes	yes	no	
139	139	S139N		DOID:2994 / germ cell cancer∣Biomuta; DOID:2394 / ovarian cancer∣Biomuta	-0.79	0.11	no	yes		
141	141	M141L	rs750876321	not provided∣ClinVar	-0.38	0.27	yes	yes	yes	
143	143	G143V		DOID:3571 / liver cancer∣Biomuta	-1.43	0.44	yes		yes	
144	144	H144P	rs1025180934	Deficiency of acetyl-CoA acetyltransferase∣ClinVar	-0.77	0.72	yes	yes	yes	

Position in UniProt	Position in PDB	Variant	dbSNP	Disease∣Source	ΔΔG	RSA	surface	4-meric interface	Non 4-meric interface (predicted)	Sites/regions
145	145	Q145E	rs120074148	Deficiency of acetyl-CoA acetyltransferase∣ClinVar; Deficiency of acetyl-CoA acetyltransferase∣DisGeNet; Ketoacidosis∣DisGeNet	-0.81	0.25	yes	yes	no	
146	146	D146N		DOID:11054 / urinary bladder cancer∣Biomuta	-0.70	0.26	yes		no	
147	147	V147L		DOID:1324 / lung cancer∣Biomuta	-0.88	0	no			
152	152	G152A	rs762991875	Deficiency of acetyl-CoA acetyltransferase∣ClinVar; Deficiency of acetyl-CoA acetyltransferase∣DisGeNet; 3-ketothiolase deficiency (3KTD) [MIM:203750]∣HUMSAVAR; not provided∣ClinVar; Ketoacidosis∣DisGeNet; Neurologic Deficits∣DisGeNet	-0.88	0	no			
154	154	E154K	rs1198149157	Deficiency of acetyl-CoA acetyltransferase∣ClinVar	-0.45	0	no			
156	156	M156I		DOID:3571 / liver cancer∣Biomuta	-1.45	0.01	no			
158	158	N158S	rs199524907	Deficiency of acetyl-CoA acetyltransferase∣ClinVar; Deficiency of acetyl-CoA acetyltransferase∣DisGeNet; Ketoacidosis∣DisGeNet; not provided∣ClinVar	-0.02	0.55	yes		yes	
158	158	N158D	rs148639841	Deficiency of acetyl-CoA acetyltransferase∣ClinVar; Deficiency of acetyl-CoA acetyltransferase∣DisGeNet; 3-ketothiolase deficiency (3KTD) [MIM:203750]∣HUMSAVAR;	-0.25	0.55	yes		yes	

Position in UniProt	Position in PDB	Variant	dbSNP	Disease∣Source	ΔΔG	RSA	surface	4-meric interface	Non 4-meric interface (predicted)	Sites/regions
171	171	G171C		DOID:1324 / lung cancer∣Biomuta	0.13	0.96	yes	yes	yes	
176	176	E176K		DOID:1612 / breast cancer∣Biomuta	-0.37	0.29	yes		yes	
176	176	E176Q		DOID:4362 / cervical cancer∣Biomuta	-0.33	0.29	yes		yes	
178	178	L178F	rs1320114846	Deficiency of acetyl-CoA acetyltransferase∣ClinVar	-0.17	0.09	no	yes		
183	183	G183R	rs120074141	Deficiency of acetyl-CoA acetyltransferase∣ClinVar; Deficiency of acetyl-CoA acetyltransferase∣DisGeNet; 3-ketothiolase deficiency (3KTD) [MIM:203750]∣HUMSAVAR; Ketoacidosis∣DisGeNet	-0.04	0.08	no	yes		
186	186	D186G		DOID:10534 / stomach cancer∣Biomuta	-0.68	0.02	no	yes		
186	186	D186Y	rs1591367592	Deficiency of acetyl-CoA acetyltransferase∣ClinVar	0.65	0.02	no	yes		
192	192	H192R		Episodic metabolic acidosis∣ClinVar; Abnormality of acetylcarnitine metabolism∣ClinVar	-0.73	0.29	yes	yes	yes	
192	192	H192N		DOID:363 / uterine cancer∣Biomuta	-0.98	0.29	yes	yes	yes	
193	193	M193R	rs541517496	Deficiency of acetyl-CoA acetyltransferase∣ClinVar	-1.32	0.09	no			
193	193	M193T	rs541517496	Deficiency of acetyl-CoA acetyltransferase∣ClinVar	-2.33	0.09	no			
201	201	A201V	rs1591368724	Deficiency of acetyl-CoA acetyltransferase∣ClinVar	-0.74	0.05	no			
203	203	K203R		DOID:1115 / sarcoma∣Biomuta	0.02	0.58	yes		yes	
208	208	R208G	rs532190594	Deficiency of acetyl-CoA acetyltransferase∣ClinVar	-1.62	0.21	yes		yes	

Position in UniProt	Position in PDB	Variant	dbSNP	Disease∣Source	ΔΔG	RSA	surface	4-meric interface	Non 4-meric interface (predicted)	Sites/regions
208	208	R208Q	rs370720208	Deficiency of acetyl-CoA acetyltransferase∣ClinVar; Deficiency of acetyl-CoA acetyltransferase∣DisGeNet; DOID:3963 / thyroid carcinoma∣Biomuta; DOID:9256 / colorectal cancer∣Biomuta; not provided∣ClinVar; DOID:363 / uterine cancer∣Biomuta; Ketoacidosis∣DisGeNet	-1.36	0.21	yes		yes	
211	211	Q211R		DOID:0070003 / blastoma∣Biomuta; DOID:3571 / liver cancer∣Biomuta	-0.58	0	no			
215	215	A215N	rs1591368794	Deficiency of acetyl-CoA acetyltransferase∣ClinVar	-1.81	0	no			
215	215	A215V		DOID:3070 / malignant glioma∣Biomuta	-0.89	0	no			
218	218	S218F	rs879255505	Deficiency of acetyl-CoA acetyltransferase∣ClinVar; Deficiency of acetyl-CoA acetyltransferase∣DisGeNet; Ketoacidosis∣DisGeNet	-0.69	0	no			
218	218	S218A		DOID:1612 / breast cancer∣Biomuta	-0.89	0	no			
219	219	Y219H	rs1437567292	Deficiency of acetyl-CoA acetyltransferase∣ClinVar	-0.30	0.05	no			Binding site
222	222	S222R	rs1591368826	Deficiency of acetyl-CoA acetyltransferase∣ClinVar	-0.23	0	no			
225	225	A225E	rs1385465985	Deficiency of acetyl-CoA acetyltransferase∣ClinVar	-1.32	0.02	no			
239	239	T239A		DOID:363 / uterine cancer∣Biomuta	0.00	0.49	yes		no	
240	240	V240D		DOID:1324 / lung cancer∣Biomuta	-2.14	0.1	no			
241	241	T241A		Deficiency of acetyl-CoA acetyltransferase∣ClinVar	-0.24	0.7	yes		yes	
247	247	D247E		DOID:2531 / hematologic cancer∣Biomuta	0.11	0.6	yes		yes	

Position in UniProt	Position in PDB	Variant	dbSNP	Disease∣Source	ΔΔG	RSA	surface	4-meric interface	Interface (predicted)	Sites/regions
252	252	E252K		Deficiency of acetyl-CoA acetyltransferase∣ClinVar	-0.21	0.29	yes		no	
253	253	D253N	rs869312946	Inborn genetic diseases∣ClinVar	-0.56	0	no			
253	253	D253E	rs983216159	Deficiency of acetyl-CoA acetyltransferase∣ClinVar	-0.18	0	no			
254	254	E254K	rs1591370241	Deficiency of acetyl-CoA acetyltransferase∣ClinVar	-0.92	0.19	no			
255	255	E255D	rs794727893	Deficiency of acetyl-CoA acetyltransferase∣ClinVar; not provided∣ClinVar	-0.71	0.07	no			
255	255	E255A	rs1591370252	Deficiency of acetyl-CoA acetyltransferase∣ClinVar	-0.48	0.07	no			
256	256	Y256S		DOID:0070003 / blastoma∣Biomuta; DOID:3571 / liver cancer∣Biomuta	-2.23	0.08	no			
256	256	Y256N	rs794727894	not provided∣ClinVar	-2.09	0.08	no			
257	257	K257T	rs370652435	Deficiency of acetyl-CoA acetyltransferase∣ClinVar	-0.47	0.54	yes		no	
258	258	R258C	rs753816946	Deficiency of acetyl-CoA acetyltransferase∣ClinVar; DOID:1909 / melanoma∣Biomuta	-0.44	0.52	yes		yes	
260	260	D260N		DOID:363 / uterine cancer∣Biomuta	-0.12	0.53	yes		yes	
262	262	S262I		DOID:3571 / liver cancer∣Biomuta	0.89	0.55	yes		yes	
262	262	S262N		DOID:363 / uterine cancer∣Biomuta	-0.03	0.55	yes		yes	
276	276	G276V		DOID:3571 / liver cancer∣Biomuta	0.25	0.14	no			
277	277	T277A		DOID:363 / uterine cancer∣Biomuta	-1.20	0.12	no			
277	277	T277P	rs1591371019	Deficiency of acetyl-CoA acetyltransferase∣ClinVar	-0.43	0.12	no			

Position in UniProt	Position in PDB	Variant	dbSNP	Disease∣Source	ΔΔG	RSA	surface	4-meric interface	Interface (predicted)	Sites/regions
282	282	N282S		DOID:0070003 / blastoma∣Biomuta; DOID:1793 / pancreatic cancer∣Biomuta	-0.65	0	no			
282	282	N282H	rs750195919	Deficiency of acetyl-CoA acetyltransferase∣ClinVar	-0.28	0	no			
283	283	A283V		DOID:263 / kidney cancer∣Biomuta	-0.44	0.05	no			
283	283	A283D		DOID:363 / uterine cancer∣Biomuta	-1.98	0.05	no			
284	284	S284N	rs1591371045	Deficiency of acetyl-CoA acetyltransferase∣ClinVar	-0.78	0.19	no			Binding site
285	285	T285I	rs1239221388	Deficiency of acetyl-CoA acetyltransferase∣ClinVar	0.30	0.2	yes		yes	
287	287	N287K	rs1351732995	not provided∣ClinVar	-0.52	0	no			
296	296	M296V		DOID:363 / uterine cancer∣Biomuta	-0.97	0	no			
297	297	T297M	rs886041122	Deficiency of acetyl-CoA acetyltransferase∣ClinVar; Deficiency of acetyl-CoA acetyltransferase∣DisGeNet; Intellectual disability∣ClinVar; 3-ketothiolase deficiency (3KTD) [MIM:203750]∣HUMSAVAR; Epilepsy∣ClinVar; not provided∣ClinVar; Ketoacidosis∣DisGeNet	-0.98	0.04	no			
297	297	T297K	rs886041122	Deficiency of acetyl-CoA acetyltransferase∣ClinVar; Deficiency of acetyl-CoA acetyltransferase∣DisGeNet; Ketoacidosis∣DisGeNet	-0.79	0.04	no			
301	301	A301V		DOID:10534 / stomach cancer∣Biomuta	-0.84	0.04	no			

Position in UniProt	Position in PDB	Variant	dbSNP	Disease∣Source	ΔΔG	RSA	surface	4-meric interface	Interface (predicted)	Sites/regions
301	301	A301P	rs1420321267	Deficiency of acetyl-CoA acetyltransferase∣ClinVar; Deficiency of acetyl-CoA acetyltransferase∣DisGeNet; 3-ketothiolase deficiency (3KTD) [MIM:203750]∣HUMSAVAR	-2.06	0.04	no			
302	302	K302N		DOID:1612 / breast cancer∣Biomuta	-0.66	0.74	yes		no	
312	312	I312T	rs120074146	Deficiency of acetyl-CoA acetyltransferase∣ClinVar; Deficiency of acetyl-CoA acetyltransferase∣DisGeNet; 3-ketothiolase deficiency (3KTD) [MIM:203750]∣HUMSAVAR; Ketoacidosis∣DisGeNet	-3.06	0.09	no			
317	317	D317E		DOID:3963 / thyroid carcinoma∣Biomuta	-0.11	0.29	yes	yes	no	
317	317	D317N	rs780486838	Deficiency of acetyl-CoA acetyltransferase∣ClinVar	-0.40	0.29	yes	yes	no	
318	318	A318T		DOID:1793 / pancreatic cancer∣Biomuta; DOID:363 / uterine cancer∣Biomuta	-0.42	0.13	no	yes		
318	318	A318S		DOID:3571 / liver cancer∣Biomuta	-0.33	0.13	no	yes		
321	321	E321D		DOID:0070003 / blastoma∣Biomuta; DOID:3571 / liver cancer∣Biomuta	0.43	0.62	yes		yes	
323	323	I323V	rs1282394804	Deficiency of acetyl-CoA acetyltransferase∣ClinVar	-0.25	0.16	no			
323	323	I323T	rs755806238	Deficiency of acetyl-CoA acetyltransferase∣ClinVar	-0.43	0.16	no			
327	327	I327V	rs150038447	Deficiency of acetyl-CoA acetyltransferase∣ClinVar	-0.56	0.16	no			

Position in UniProt	Position in PDB	Variant	dbSNP	Disease∣Source	ΔΔG	RSA	surface	4-meric interface	Interface (predicted)	Sites/regions
328	328	A328V	rs1057517702	not specified∣ClinVar	-0.82	0	no			
333	333	A333P	rs120074147	Deficiency of acetyl-CoA acetyltransferase∣ClinVar; Deficiency of acetyl-CoA acetyltransferase∣DisGeNet; 3-ketothiolase deficiency (3KTD) [MIM:203750]∣HUMSAVAR; Ketoacidosis∣DisGeNet	-1.79	0	no			
334	334	S334F		DOID:363 / uterine cancer∣Biomuta	-0.37	0.4	yes		yes	
335	335	M335I		DOID:3571 / liver cancer∣Biomuta	-0.68	0.48	yes	yes	yes	
337	337	L337R		DOID:1612 / breast cancer∣Biomuta	-1.93	0.08	no			
337	337	L337F		DOID:1324 / lung cancer∣Biomuta	-0.86	0.08	no			
341	341	G341R		DOID:1909 / melanoma∣Biomuta	-0.18	0.8	yes		no	
347	347	I347T	rs1338023969	Deficiency of acetyl-CoA acetyltransferase∣ClinVar	-2.93	0	no			
350	350	W350C		DOID:3571 / liver cancer∣Biomuta	-2.16	0.01	no			
353	353	N353K	rs1591374629	Deficiency of acetyl-CoA acetyltransferase∣ClinVar	-0.47	0.01	no			
354	354	E354V	rs1591374632	Deficiency of acetyl-CoA acetyltransferase∣ClinVar	0.15	0	no			
355	355	A355V		DOID:1793 / pancreatic cancer∣Biomuta	-0.77	0.07	no			
357	357	S357C		DOID:4362 / cervical cancer∣Biomuta	-0.46	0	no			
357	357	S357I		DOID:3571 / liver cancer∣Biomuta	0.09	0	no			
362	362	A362T		DOID:10534 / stomach cancer∣Biomuta	-1.10	0	no			
364	364	I364T		DOID:10534 / stomach cancer∣Biomuta	-1.66	0.15	no			
365	365	K365R		DOID:1324 / lung cancer∣Biomuta	0.15	0.48	yes		yes	
369	369	I369V	rs1591374695	Deficiency of acetyl-CoA acetyltransferase∣ClinVar	-0.61	0.08	no			
372	372	Q372K		DOID:3963 / thyroid carcinoma∣Biomuta	-0.04	0.65	yes		no	

Position in UniProt	Position in PDB	Variant	dbSNP	Disease∣Source	ΔΔG	RSA	surface	4-meric interface	Interface (predicted)	Sites/regions
375	375	N375S	rs373771053	Deficiency of acetyl-CoA acetyltransferase∣ClinVar; Deficiency of acetyl-CoA acetyltransferase∣DisGeNet	-0.83	0.01	no			
377	377	N377T		DOID:1612 / breast cancer∣Biomuta	-0.36	0.05	no			
379	379	G379V	rs120074143	Deficiency of acetyl-CoA acetyltransferase∣ClinVar; Deficiency of acetyl-CoA acetyltransferase∣DisGeNet; 3-ketothiolase deficiency (3KTD) [MIM:203750]∣HUMSAVAR; Ketoacidosis∣DisGeNet	-1.32	0.01	no			
379	379	G379A		DOID:11054 / urinary bladder cancer∣Biomuta	-1.04	0.01	no			
380	380	A380T	rs120074140	Deficiency of acetyl-CoA acetyltransferase∣ClinVar; Deficiency of acetyl-CoA acetyltransferase∣DisGeNet; 3-ketothiolase deficiency (3KTD) [MIM:203750]∣HUMSAVAR; Ketoacidosis∣DisGeNet; Neurologic Deficits∣DisGeNet	-0.71	0	no			
380	380	A380S		DOID:10283 / prostate cancer∣Biomuta	-0.47	0	no			
381	381	V381A		DOID:9256 / colorectal cancer∣Biomuta; DOID:1909 / melanoma∣Biomuta	-1.51	0	no			
382	382	S382F		DOID:11054 / urinary bladder cancer∣Biomuta	0.21	0	no			

Position in UniProt	Position in PDB	Variant	dbSNP	Disease∣Source	ΔΔG	RSA	surface	4-meric interface	Interface (predicted)	Sites/regions
387	387	I387T	rs748303093	Deficiency of acetyl-CoA acetyltransferase∣ClinVar; Deficiency of acetyl-CoA acetyltransferase∣DisGeNet; not provided∣ClinVar; Ketoacidosis∣DisGeNet; Neurologic Deficits∣DisGeNet	-2.17	0.08	no			
388	388	G388W		DOID:1324 / lung cancer∣Biomuta	-0.31	0	no			
388	388	G388E	rs773491386	Deficiency of acetyl-CoA acetyltransferase∣ClinVar	-1.20	0	no			
388	388	G388A		DOID:0070003 / blastoma∣Biomuta; DOID:3571 / liver cancer∣Biomuta	-0.53	0	no			
389	389	M389I	rs377295639	Deficiency of acetyl-CoA acetyltransferase∣ClinVar	-1.26	0	no			
390	390	S390P	rs1184088336	Deficiency of acetyl-CoA acetyltransferase∣ClinVar	-1.33	0	no			
397	397	H397Q		DOID:10283 / prostate cancer∣Biomuta	-0.73	0.02	no			
397	397	H397D	rs746332363	Deficiency of acetyl-CoA acetyltransferase∣ClinVar	-1.28	0.02	no			
400	400	H400Y	rs201183545	Deficiency of acetyl-CoA acetyltransferase∣ClinVar	-0.04	0.27	yes		no	
400	400	H400R	rs761086326	Deficiency of acetyl-CoA acetyltransferase∣ClinVar	-0.51	0.27	yes		no	
406	406	E406G	rs147872303	Deficiency of acetyl-CoA acetyltransferase∣ClinVar	-0.71	0.31	yes		no	
407	407	Y407C		DOID:9256 / colorectal cancer∣Biomuta	-0.94	0.23	yes		no	
408	408	G408V	rs1131691780	not provided∣ClinVar	1.27	0	no			
408	408	G408R	rs553101581	Deficiency of acetyl-CoA acetyltransferase∣ClinVar	0.06	0	no			

Position in UniProt	Position in PDB	Variant	dbSNP	Disease∣Source	ΔΔG	RSA	surface	4-meric interface	Interface (predicted)	Sites/regions
410	410	A410V	rs767412638	Deficiency of acetyl-CoA acetyltransferase∣ClinVar	0.32	0	no			
416	416	G416E		DOID:4362 / cervical cancer∣Biomuta	-0.17	0.38	yes	yes	no	
417	417	G417E		DOID:4362 / cervical cancer∣Biomuta	-0.44	0.01	no			
418	418	G418D	rs886042080	Deficiency of acetyl-CoA acetyltransferase∣ClinVar; not provided∣ClinVar	-0.35	0.07	no			
421	421	A421S		DOID:3571 / liver cancer∣Biomuta	-0.55	0	no			
421	421	A421T		DOID:1793 / pancreatic cancer∣Biomuta	-0.50	0	no			
421	421	A421V		DOID:0070003 / blastoma∣Biomuta; DOID:3571 / liver cancer∣Biomuta	0.26	0	no			
423	423	L423R		DOID:3571 / liver cancer∣Biomuta	-1.24	0	no			
427	427	L427M		Deficiency of acetyl-CoA acetyltransferase∣ClinVar	-0.12	0.52	yes		no	

Variants in ACAT1 are derived from different sources including Humsavar, OMIM, ClinVar, Biomuta. Variants are mapped on the PDB file 2IBY, chain A. ΔΔG values are predicted with INPS starting from structure. RSA= Relative Solvent Accessibility computed with DSSP. Surface residues are those with RSA ≥ 0.2. Residues that are part of the tetrameric interface are computed from the Biological Assembly of 2IBY. Residues in non-tetrameric interfaces are predicted with ISPRED4. Sites and regions are derived from UniProt annotations.

Supplementary Table S4: *Variants in ACAT2*

Position in UniProt	Position in PDB	Variant	dbSNP	Disease∣Source	ΔΔG	RSA	surface	4-meric interface	Non 4-meric interface (predicted)	Sites/regions
3	-	A3S		DOID:11054 / urinary bladder cancer∣Biomuta						
9	9	V9F		DOID:3571 / liver cancer∣Biomuta	-0.53	0	no			
13	13	A13V		DOID:3571 / liver cancer∣Biomuta	0.45	0	no			
15	15	R15L		DOID:3571 / liver cancer∣Biomuta	0.76	0	no			
21	21	F21L		DOID:4362 / cervical cancer∣Biomuta	-0.58	0.42	yes	yes	no	
22	22	N22S		DOID:2994 / germ cell cancer∣Biomuta	0.08	0.72	yes		no	
26	26	A26D		DOID:363 / uterine cancer∣Biomuta	-0.77	0.22	yes		no	
31	31	Q31H		DOID:363 / uterine cancer∣Biomuta	-0.61	0.11	no			
34	34	G34A		DOID:0060119 / pharynx cancer∣Biomuta	0.65	0	no			
42	42	L42F		DOID:1324 / lung cancer∣Biomuta	-1.12	0.05	no			
49	49	P49L		DOID:363 / uterine cancer∣Biomuta	-0.46	0.3	yes		no	
52	52	V52M		DOID:0070003 / blastoma∣Biomuta; DOID:3571 / liver cancer∣Biomuta	-1.46	0.04	no			
58	58	G58V		DOID:3571 / liver cancer∣Biomuta	-0.81	0	no			
62	62	A62S		DOID:1324 / lung cancer∣Biomuta	-0.05	0.42	yes		yes	
73	73	A73S		DOID:3571 / liver cancer∣Biomuta	-0.59	0	no			
78	78	G78E		DOID:1909 / melanoma∣Biomuta	-1.15	0.55	yes		no	
80	80	P80S		DOID:1909 / melanoma∣Biomuta	-0.95	0.32	yes		no	
83	83	V83F		DOID:0070003 / blastoma∣Biomuta; DOID:3571 / liver cancer∣Biomuta	-1.78	0.04	no			
93	93	G93W		DOID:3571 / liver cancer∣Biomuta	-0.25	0.07	no			
106	106	I106M		DOID:1909 / melanoma∣Biomuta	-1.63	0	no			
108	108	I108V		DOID:3571 / liver cancer∣Biomuta	0.38	0.75	yes		yes	
113	113	I113M		DOID:1324 / lung cancer∣Biomuta	-0.43	0.01	no			
121	121	N121H		DOID:1324 / lung cancer∣Biomuta	-0.23	0.04	no			

Position in UniProt	Position in PDB	Variant	dbSNP	Disease∣Source	ΔΔG	RSA	surface	4-meric interface	Non 4-meric interface (predicted)	Sites/regions
122	122	M122T		DOID:10534 / stomach cancer∣Biomuta	-2.18	0.01	no			
134	134	G134E		DOID:263 / kidney cancer∣Biomuta	-0.06	0.61	yes	yes	yes	
139	139	E139D		DOID:11934 / head and neck cancer∣Biomuta; DOID:363 / uterine cancer∣Biomuta	-0.08	0.98	yes	yes	yes	
147	147	L147I		DOID:0070003 / blastoma∣Biomuta; DOID:3571 / liver cancer∣Biomuta	-0.12	0.5	yes		yes	
147	147	L147P		DOID:363 / uterine cancer∣Biomuta	-0.87	0.5	yes		yes	
148	148	C148S		DOID:0070003 / blastoma∣Biomuta; DOID:3571 / liver cancer∣Biomuta	-0.79	0.43	yes	yes	yes	
153	153	D153G		DOID:0070003 / blastoma∣Biomuta; DOID:3571 / liver cancer∣Biomuta	-0.69	0.02	no			
158	158	C158G		DOID:363 / uterine cancer∣Biomuta	-1.30	0.17	no			
160	160	M160I		DOID:1793 / pancreatic cancer∣Biomuta	-0.61	0.1	no	yes		
161	161	G161S		DOID:3070 / malignant glioma∣Biomuta	-0.22	0	no			
176	176	E176K	rs371307389	Acetyl-CoA acetyltransferase-2 deficiency∣ClinVar	-0.12	0.6	yes		yes	
179	179	D179H		DOID:0070003 / blastoma∣Biomuta; DOID:3571 / liver cancer∣Biomuta	-0.19	0.02	no			
184	184	L184V		DOID:1612 / breast cancer∣Biomuta	-1.09	0.48	yes		yes	
192	192	A192S		DOID:3571 / liver cancer∣Biomuta	-0.90	0.02	no			
195	195	A195V		DOID:0070003 / blastoma∣Biomuta; DOID:3571 / liver cancer∣Biomuta	-1.39	0.59	yes		no	
195	195	A195S		DOID:3070 / malignant glioma∣Biomuta	-0.49	0.59	yes		no	
197	197	H197R		DOID:2531 / hematologic cancer∣Biomuta	-0.24	0.29	yes		no	
203	203	V203E		DOID:1612 / breast cancer∣Biomuta	-1.38	0.07	no			
210	210	R210K		DOID:5041 / esophageal cancer∣Biomuta	-0.16	0.88	yes		yes	

Position in UniProt	Position in PDB	Variant	dbSNP	Disease∣Source	ΔΔG	RSA	surface	4-meric interface	Non 4-meric interface (predicted)	Sites/regions
211	211	K211R	rs25683	Polymorphism∣HUMSAVAR; DOID:9256 / colorectal cancer∣Biomuta	0.37	0.98	yes		no	
219	219	D219Y		DOID:3571 / liver cancer∣Biomuta	0.72	0.06	no			
219	219	D219H		DOID:9256 / colorectal cancer∣Biomuta	-0.21	0.06	no			
221	221	F221C		DOID:363 / uterine cancer∣Biomuta	-1.17	0.26	yes		yes	
237	237	Y237C		DOID:363 / uterine cancer∣Biomuta	-0.23	0.67	yes		yes	
243	243	T243M		DOID:3070 / malignant glioma∣Biomuta; DOID:9256 / colorectal cancer∣Biomuta	0.22	0.58	yes		yes	
243	243	T243A		DOID:10534 / stomach cancer∣Biomuta	-0.27	0.58	yes		yes	
262	262	V262I		DOID:363 / uterine cancer∣Biomuta	-0.34	0	no			
270	270	D270N		DOID:0070003 / blastoma∣Biomuta	-0.28	0.53	yes		no	
272	272	R272C		DOID:363 / uterine cancer∣Biomuta	-0.77	0.32	yes		no	
274	274	L274R		DOID:1793 / pancreatic cancer∣Biomuta	-1.23	0.13	no			
278	278	A278V		DOID:10534 / stomach cancer∣Biomuta	-0.04	0	no			
279	279	R279Q		DOID:1324 / lung cancer∣Biomuta	-0.98	0.31	yes		no	
280	280	I280V		DOID:9256 / colorectal cancer∣Biomuta	-0.58	0.08	no			
284	284	S284C		not provided∣ClinVar	-0.25	0.01	no			
286	286	V286A		DOID:11054 / urinary bladder cancer∣Biomuta	-0.35	0.12	no			
292	292	I292V		DOID:9256 / colorectal cancer∣Biomuta; DOID:1909 / melanoma∣Biomuta	-0.38	0.16	no			
300	300	A300T		DOID:1324 / lung cancer∣Biomuta	-1.09	0	no			
304	304	A304S		DOID:3963 / thyroid carcinoma∣Biomuta	-1.39	0	no			
310	310	W310L		DOID:3571 / liver cancer∣Biomuta	-0.33	0.13	no			
329	329	A329S		DOID:9256 / colorectal cancer∣Biomuta; DOID:1909 / melanoma∣Biomuta	-0.76	0.17	no			
335	335	L335V		DOID:3070 / malignant glioma∣Biomuta	-1.62	0.13	no			

Position in UniProt	Position in PDB	Variant	dbSNP	Disease∣Source	ΔΔG	RSA	surface	4-meric interface	Non 4-meric interface (predicted)	Sites/regions
336	336	G336R		DOID:11054 / urinary bladder cancer∣Biomuta	-0.68	0.62	yes		no	
341	341	K341R		DOID:0070003 / blastoma∣Biomuta; DOID:3571 / liver cancer∣Biomuta	-0.76	0.18	no			
358	358	S358F		DOID:363 / uterine cancer∣Biomuta	-0.62	0	no			
363	363	L363I		DOID:363 / uterine cancer∣Biomuta; DOID:9256 / colorectal cancer∣Biomuta; DOID:1909 / melanoma∣Biomuta	-0.45	0	no			
363	363	L363V		DOID:1324 / lung cancer∣Biomuta	-0.73	0	no			
365	365	T365I		DOID:3070 / malignant glioma∣Biomuta	0.10	0	no			
371	371	E371Q		DOID:1324 / lung cancer∣Biomuta	-0.17	0.35	yes		no	
376	376	S376I		DOID:3571 / liver cancer∣Biomuta	0.01	0.25	yes		no	
377	377	R377C		DOID:11934 / head and neck cancer∣Biomuta; DOID:9256 / colorectal cancer∣Biomuta; DOID:363 / uterine cancer∣Biomuta	-0.46	0.31	yes		no	
380	380	A380V		DOID:10534 / stomach cancer∣Biomuta	0.75	0	no			
385	385	G385A		DOID:0070003 / blastoma∣Biomuta; DOID:3571 / liver cancer∣Biomuta	-0.07	0.12	no			
387	387	G387V		DOID:0070003 / blastoma∣Biomuta; DOID:3571 / liver cancer∣Biomuta	0.68	0.02	no	yes		
388	388	M388V		DOID:9256 / colorectal cancer∣Biomuta	-0.40	0.34	yes	yes	yes	
394	394	V394A		DOID:9256 / colorectal cancer∣Biomuta	-1.19	0	no			
394	394	V394F		DOID:3571 / liver cancer∣Biomuta	-0.41	0	no			

Variants in ACAT2 are derived from different sources including Humsavar, OMIM, ClinVar, Biomuta. Variants are mapped on the PDB file 1WL4, chain A. ΔΔG values are predicted with INPS starting from structure. RSA= Relative Solvent Accessibility computed with DSSP. Surface residues are those with RSA ≥ 0.2. Residues that are part of the

tetrameric interface are computed from the Biological Assembly of 1WL4. Residues in non-tetrameric interfaces are predicted with ISPRED4. Sites and regions are derived from UniProt annotations.
